# Cypripedin Induces Apoptosis and Synergizes with Bortezomib via ER Stress Mediated Ubiquitination of GRP78 in T-Cell Acute Lymphoblastic Leukemia

**DOI:** 10.3390/molecules31111823

**Published:** 2026-05-25

**Authors:** Zin Zin Ei, Bodee Nutho, Boonchoo Sritularak, Pithi Chanvorachote, Preedakorn Chunhacha

**Affiliations:** 1 Department of Pharmacology and Physiology, Faculty of Pharmaceutical Sciences, Chulalongkorn University, Bangkok 10330, Thailand; zinzin.e@chula.ac.th (Z.Z.E.);; 2 Center of Excellence in Cancer Cell and Molecular Biology, Faculty of Pharmaceutical Sciences, Chulalongkorn University, Bangkok 10330, Thailand; 3 Department of Pharmacology, Faculty of Science, Mahidol University, Bangkok 10400, Thailand; bodee.nut@mahidol.ac.th; 4 Department of Pharmacognosy and Pharmaceutical Botany, Faculty of Pharmaceutical Sciences, Chulalongkorn University, Bangkok 10330, Thailand; 5 Center of Excellence in Natural Products for Ageing and Chronic Diseases, Faculty of Pharmaceutical Sciences, Chulalongkorn University, Bangkok 10330, Thailand; 6 Department of Biochemistry and Microbiology, Faculty of Pharmaceutical Sciences, Chulalongkorn University, Bangkok 10330, Thailand

**Keywords:** cypripedin, Jurkat cells, T-ALL, apoptosis, bortezomib, endoplasmic reticulum stress, GRP78, ubiquitination

## Abstract

Background: T-cell acute lymphoblastic leukemia (T-ALL) remains a challenging malignancy with limited targeted therapies. Natural phenanthrene derivatives represent a promising source of antileukemic agents. Objective: We screened a library of natural phenanthrene-type compounds to identify cytotoxic leads in Jurkat T-ALL cells and investigated the mechanisms underlying their activity, including potential synergy with the proteasome inhibitor bortezomib (BTZ). Methods: Jurkat cells were treated with thirteen natural compounds at 10 and 20 µM for 48 h; cell viability was assessed by WST-1 cell viability assay. Dose–response curves were generated to calculate IC_50_ values. Apoptosis was evaluated by Hoechst 33342/PI staining and Annexin V/PI flow cytometry. Synergy with BTZ was analyzed using a fixed-ratio combination index (CI) approach and IC_50_ shift analysis. ER stress signaling was characterized by Western blotting, quantitative RT-PCR of UPR genes (GRP78, ATF6), and immunoprecipitation of GRP78 followed by ubiquitin immunoblotting. Results: Among the compounds screened, Cypripedin showed the most potent cytotoxicity with an IC_50_ of 6.52 µM. It induced a dose-dependent increase in apoptosis. Combination with BTZ yielded a CI < 0.5 and reduced BTZ IC_50_ from 3.43 to 1.88 ng/mL. Cypripedin activated the unfolded protein response (UPR), modulated key ER stress markers including GRP78, p-PERK, p-eIF2α, p-JNK, and ATF6, downregulated UPR gene transcripts, and promoted GRP78 ubiquitination. Molecular docking predicted strong binding of Cypripedin to the GRP78 ATPase domain (Vina score −7.630 kcal/mol), supporting its mechanism of action. Conclusion: Cypripedin induces apoptosis in Jurkat T-ALL cells, synergizes with BTZ, and modulates ER stress through GRP78 ubiquitination. These findings support its further development as a potential T-ALL therapeutic.

## 1. Introduction

T-cell acute lymphoblastic leukemia (T-ALL) is an aggressive hematologic malignancy, accounting for approximately 15% of pediatric and 25% of adult acute lymphoblastic leukemia cases [1]. Despite advances in combination chemotherapy, the long-term survival rate for patients with relapsed or refractory T-ALL remains below 10% [2], underscoring the urgent need for novel therapeutic strategies that can overcome drug resistance while minimizing systemic toxicity.

Proteasome inhibitors such as bortezomib (BTZ) have demonstrated efficacy in T-ALL by promoting the accumulation of misfolded proteins, thereby inducing endoplasmic reticulum (ER) stress and triggering apoptosis [3]. Preclinical data indicate that BTZ exhibits only modest single-agent cytotoxicity ex vivo in T-ALL models but shows enhanced efficacy when combined with agents such as dexamethasone or histone deacetylase (HDAC) inhibitors, suggesting that BTZ monotherapy is insufficient [4].

ER stress activates the unfolded protein response (UPR), a conserved adaptive signaling network mediated by three principal sensors: protein kinase RNA-like ER kinase (PERK), inositol-requiring enzyme 1 (IRE1), and activating transcription factor 6 (ATF6). Upon ER stress, GRP78 dissociates from these sensors, resulting in the activation of downstream pathways, including PERK-mediated phosphorylation of eIF2α, IRE1-dependent activation of stress kinases such as JNK, ATF6 cleavage and transcriptional regulation of ER chaperones. These pathways collectively determine cell fate by balancing adaptive responses and apoptotic signaling.

In multiple myeloma, BTZ-induced ER stress resulting from misfolded protein accumulation activates a GRP78 (78 kDa glucose-regulated protein)-mediated unfolded protein response (UPR) that promotes cell survival. However, targeting GRP78 disrupts this adaptive mechanism and enhances the cytotoxicity of proteasome inhibitors—a vulnerability that has been exploited in combination therapy strategies. A 2023 study demonstrated that combining the GRP78 inhibitor HA15 with BTZ significantly increased ER stress markers (GRP78, ATF4, CHOP, XBP1), inhibited colony formation, and enhanced apoptosis compared to BTZ alone. Importantly, this combination more effectively suppressed tumor growth in vivo, confirming that HA15 sensitizes cells to proteasome inhibition [5]. However, the therapeutic potential of modulating GRP78 in T-ALL remains largely unexplored.

Natural products have historically served as a prolific source of anticancer agents [6]. Among these, phenanthrene-type alkaloids and related compounds derived from medicinal orchids exhibit diverse bioactivities, including cytotoxicity against solid tumors [7]. Phenanthrenoid alkaloids, characterized by their rigid tricyclic architectures analogous to anthracycline antibiotics [8], are emerging as modulators of endoplasmic reticulum (ER) stress in preclinical cancer models [9,10,11]. However, their potential in hematologic malignancies and the underlying mechanisms of action remain underexplored. In particular, the ability of such compounds to disrupt ER homeostasis and modulate chaperone function has not been systematically investigated in T-ALL models.

Here, we performed a medium-throughput screen of thirteen naturally derived phenanthrene-type compounds to evaluate their cytotoxic effects in the Jurkat T-ALL cell line. Cypripedin was identified as the most potent compound, exhibiting low-micromolar IC_50_ values. Its pro-apoptotic activity was subsequently characterized using complementary assays. We further evaluated the potential synergy between Cypripedin and the proteasome inhibitor bortezomib, which revealed strong synergism by Chou–Talalay analysis and a twofold reduction in the IC_50_ of bortezomib.

To elucidate the mechanistic basis of these effects, we examined ER stress activation through key unfolded protein response (UPR) markers, including GRP78, PERK/eIF2α, JNK, and ATF6. We also assessed the transcriptional regulation of UPR-related genes and demonstrated that Cypripedin is associated with increased ubiquitination of GRP78. Finally, in silico molecular docking supported these findings by predicting direct binding of Cypripedin to the ATPase cleft of GRP78. This integrated approach identifies Cypripedin as a novel natural compound that engages ER stress signaling to induce apoptosis and enhance the cytotoxic effects of proteasome inhibition in T-ALL cells.

## 2. Materials and Methods

### 2.1. Cell Culture and Reagents

Jurkat T-ALL cells (accession number CVCL_0367, Homo sapiens) were acquired from the American Type Culture Collection (ATCC, Manassas, VA, USA). These cells were cultured in RPMI 1640 medium (Cat no. 11875-093, Gibco, Grand Island, NY, USA), supplemented with 10% fetal bovine serum (FBS; Cat no. SV30160.03, HyClone™, Cytiva, Global Life Sciences, Vienna, Austria), 2 mM L-glutamine (Ref no. 35050-061, Gibco, Gaithersburg, MA, USA), and 100 U/mL of antibiotic–antimycotic (Ref no. 15240-062, Gibco, Grand Island, NY, USA). Cells were incubated at 37 °C in a humidified atmosphere containing 5% CO_2_.

All cell culture reagents, including RPMI 1640 medium, L-glutamine, FBS, antibiotic–antimycotic, and phosphate-buffered saline (PBS), were purchased from Gibco (Grand Island, NY, USA). Additional reagents used in the study included Cell Proliferation Reagent WST-1 (Cat no. 05 015 944 001), bovine serum albumin (BSA; Cat no. 9048-46-8), Tunicamycin (product no: 654380), Hoechst 33342 (Cat no. B2261), and propidium iodide (PI; Cat no. P4170), all obtained from Sigma-Aldrich (St. Louis, MO, USA). MG132 (cat no: 2194S) was purchased from cell signaling. Bortezomib (Cat no: sc-217785 was obtained from Santa Cruz Biotechnology, Inc., Dallas, TX, USA.)

Primary antibodies for ER stress-related proteins were purchased from Cell Signaling Technology (Beverly, MA, USA), including rabbit monoclonal antibodies against PERK (140 kDa, Cat no: 5683), Elf2α (38 kDa, Cat no: 9722S), phospho-Elf2α (38 kDa, Cat no: 3597S), JNK (46, 54 kDa, Cat no: 9252S), phospho-JNK (46, 54 kDa, Cat no: 9255S), and ATF-6 (D4Z8V) (100 kDa, Cat no: 65880). Additional antibodies, including GRP78 (78 kDa, Cat no: ab21685) and ubiquitin (Cat no: ab134953), were obtained from Abcam (Waltham, MA, USA).

### 2.2. Extraction and Structure Elucidation of Cypripedin

**Scheme 1:**
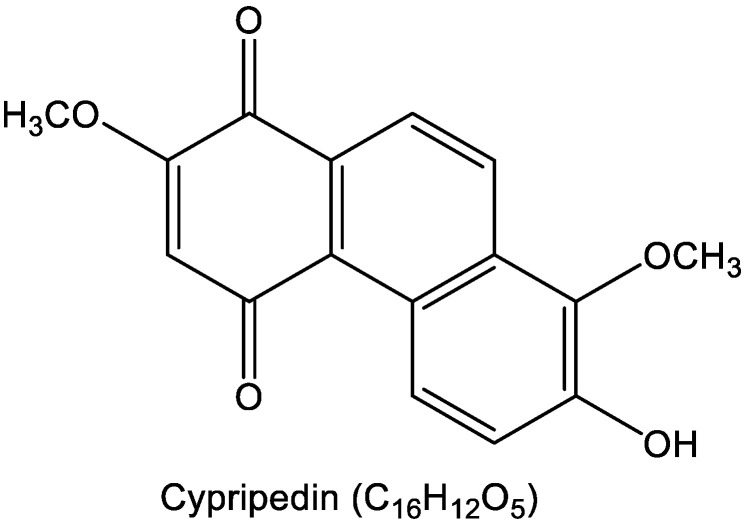
Chemical structure of cypripedin (C_16_H_12_O_5_).

Cypripedin was extracted from Dendrobium densiflorum using methanol and subsequently purified via C-18 column chromatography with an H_2_O-MeOH gradient (as described by Treesuwan S. et al. [12]. Briefly, its structure was confirmed by NMR analysis (Scheme 1), and its purity verified by both HPLC and NMR exceeded 95%, the material used in this study. Spectral data of cypripedin: The structure of cypripedin was elucidated using NMR analysis performed on a Bruker Avance DPX-300 FT-NMR (Billerica, MA, USA) spectrometer, and its mass spectrum was obtained with a Bruker microTOF mass spectrometer (ESI-MS, Billerica, MA, USA); C_16_H_12_O_5_; HR-ESI-MS [M+Na]^+^ at *m/z* 307.0582 (calcd. for 307.0582, C_16_H_12_O_5_Na); ^1^H NMR (300 MHz, acetone-*d*_6_) δ: 9.27 (1H, d, *J* = 9.6 Hz, H-5), 8.37 (1H, d, *J* = 9.0 Hz, H-9), 8.09 (1H, d, *J* = 9.0 Hz, H-10), 7.43 (1H, d, *J* = 9.6 Hz, H-6), 6.20 (1H, s, H-3), 3.94 (3H, s, MeO-8), 3.93 (3H, s, MeO-2); ^13^C NMR (75 MHz, acetone-*d*_6_) δ: 189.1 (C-4), 181.2 (C-1), 159.6 (C-2), 149.5 (C-7), 141.1 (C-8), 134.0 (C-8a), 130.0 (C-10a), 128.3 (C-4a), 127.2 (C-9), 126.2 (C-5), 125.7 (C-4b), 123.4 (C-6), 122.7 (C-10), 111.9 (C-3), 61.5 (MeO-8), 56.7 (MeO-2).

### 2.3. Preparation of Stock Solutions of Natural Products

Thirteen bioactive compounds were obtained from Assoc. Prof. Boonchoo Sritularak, which have been previously identified. The natural products were dissolved in dimethyl sulfoxide (DMSO) to prepare 50 mM stock solutions and subsequently stored at −20 °C. The DMSO concentration used in all experiments was kept below 0.1%, a level that exhibited no cytotoxic effects.

### 2.4. WST1 Cell Proliferation Assay

A cell viability assay was conducted to assess the cytotoxic effects of the natural products on Jurkat T-ALL cells. Jurkat cells were seeded at a density of 1 × 10^5^ cells per well (100 µL) in 96-well plates and incubated under 5% CO_2_ conditions. Cells were then treated with various concentrations of natural products for 24 h. Following treatment, 10 µL of WST-1 cell proliferation reagent was added to each well containing 100 µL of cell suspension. After a 2.5 h incubation with WST-1, absorbance was measured using a microplate reader. The absorbance of the formazan product was recorded within the peak absorption of 450 nm, and a reference wavelength above 600 nm was used. Relative cell viability for each treatment group was determined by comparing it to the untreated control group.

### 2.5. Nuclear Staining Assay

Apoptotic and necrotic cell death were assessed through dual staining with Hoechst 33,342 and propidium iodide (PI). Jurkat T-ALL cells were plated in 96-well plates at a density of 1  ×  10^5^ cells per well (100 µL) and incubated at 37 °C. The cells were then exposed to different concentrations of Cypripedin (0–8 µM) for 24 h. Following treatment, the cells were co-stained with Hoechst 33342 (10 µg/mL) and PI (0.02 µg/mL) for 30 min at 37 °C. Apoptotic cells showing nuclear condensation and fragmentation were detected with Hoechst 33342, while necrotic cells were identified by PI-positive staining. Stained cells were observed and imaged using a fluorescence microscope (Olympus IX51 equipped with a DP70 digital camera, Olympus, Tokyo, Japan).

### 2.6. Apoptosis Assay by Annexin V Staining

Apoptosis at early and late stages, along with necrotic cell death, was evaluated using a fluorescein isothiocyanate (FITC)-Annexin V/propidium iodide (PI) apoptosis detection kit, following the manufacturer’s protocol (ImmunoTools, catalog no. 3149001, Friesoythe, Germany). Cells were incubated with Annexin V-FITC and PI in binding buffer for 15 min at RT. The stained cells were then analyzed by flow cytometry using the Benchtop Guava^®^ easyCyte HT system operated with guavaSoft™ software (version 3.3, EMD Millipore, Billerica, MA, USA).

### 2.7. Drug Combination Analysis

Initially, the IC_50_ values of Cypripedin and Bortezomib were determined individually using the WST-1 cell proliferation assay. Following this, a combination treatment analysis was conducted to assess potential synergistic effects. Cypripedin and Bortezomib were administered both individually and in combination at a fixed ratio derived from their respective dose–response curves. The combination index (CI) was calculated using CompuSyn software (version 1.0; T.C. Chou and N. Martin, Memorial Sloan-Kettering Cancer Center, New York, NY, USA), and an isobologram was generated to quantitatively evaluate the nature of the drug interaction.

### 2.8. Western Blot Analysis

Jurkat T-ALL cells were seeded in 6-well plates at a density of 1  ×  10^6^ cells per well (1 mL) and treated with Cypripedin at concentrations ranging from 0 to 8 µM for 24 h at 37 °C. After treatment, cells were collected and washed with ice-cold PBS. Lysis was performed using a buffer containing 50 mM HEPES (pH 7.5), 150 mM NaCl, 5 mM EDTA, 1% Triton X-100, 1 mM PMSF, and 2 µg/mL pepstatin A, supplemented with a complete protease inhibitor cocktail (EASYpack, Roche, cat no: 04693116001, Mannheim, Germany). The cells were lysed on ice for 40 min. Lysates were then centrifuged at 12,000× *g* for 15 min at 4 °C, and protein concentrations were measured using the BCA protein assay kit (Thermo-Fisher Scientific, Rockford, IL, USA).

Equal amounts of protein were mixed with loading buffer and denatured by heating at 95 °C for 5 min. Proteins were separated by SDS-PAGE and transferred onto 0.45 µm nitrocellulose membranes. Membranes were blocked with 5% non-fat milk in TBST (25 mM Tris-HCl, pH 7.5, 125 mM NaCl, 0.05% Tween-20) for 1 h at room temperature, then incubated overnight at 4 °C with primary antibodies diluted 1:1000 in 5% BSA in TBST. β-Actin was used as a loading control.

The following day, membranes were washed three times with TBST (5 min each) and then incubated with HRP-conjugated secondary antibodies (anti-rabbit or anti-mouse IgG, diluted 1:2000 in 5% skim milk in TBST) for 1 h at room temperature. After further washing with TBST, protein bands were visualized using a chemiluminescent substrate (SuperSignal West Pico, Pierce, Rockford, IL, USA) then subjected to the X-ray film exposure. The molecular weight were compared against the marker (Biotinylated Protein Ladder Detection Pack^TM^, Cell Signaling Technology, cat no. 7727, Danvers, MA, USA). Signal intensity was measured by densitometry using ImageJ software (version 1.52a, National Institutes of Health, Bethesda, MD, USA).

### 2.9. Quantitative Analysis for Real-Time PCR Analysis

Total RNA was isolated from Jurkat T-ALL cells treated with Cypripedin (1 × 10^6^ cells per well in 6-well plates) using GENEzol reagent. The extracted RNA was then used for complementary DNA (cDNA) synthesis with SuperScript III reverse transcriptase (Invitrogen, Carlsbad, CA, USA). Reverse transcription quantitative PCR (RT-qPCR) was conducted using 100 ng of cDNA in a 20 µL reaction volume with Luna^®^ Universal qPCR Master Mix (New England Biolabs, Ipswich, MA, USA). Amplification was performed on a CFX96 Real-Time PCR system (Bio-Rad, Hercules, CA, USA). The thermal cycling conditions included an initial denaturation at 95 °C for 1 min, followed by 45 cycles of denaturation at 95 °C for 15 s and annealing at 60 °C for 30 s. A melting curve analysis was performed at the end of the run to confirm primer specificity. The targeted gene of primers are:

GRP78 (Fwd) GTTCTTCAATGGCAAGGAACCATC Tm = 63.5 °C

GRP78 (Rev) CCATCCTTTCGATTTCTTCAGGTG Tm = 63.5 °C

ATF 6 (Fwd) GCCTTTATTGCTTCCAGCAG Tm = 54.5 °C

ATF 6 (Rev) TGAGACAGCAAAACCGTCTG Tm = 55.5 °C

GAPDH (Fwd) GCTCAGAACACCTATGGGGAA Tm = 59.8 °C

GAPDH (Rev) CATCGCCCCACTTGATTTGG Tm =  59.8 °C

The PCR products were normalized against GAPDH, which served as internal control. Relative mRNA expression levels of the target genes were calculated using the comparative Cq (ΔΔCq) method.

### 2.10. Immunoprecipitation

Jurkat T-ALL cells treated with Cypripedin were harvested using cold PBS and lysed on ice with a buffer containing 50 mM HEPES (pH 7.5), 150 mM NaCl, 5 mM EDTA, 1% Triton X-100, 1 mM PMSF, and 2 μg/mL pepstatin A (catalog no. 9803, Cell Signaling, Danvers, MA, USA), supplemented with a complete protease inhibitor cocktail (EASYpack, Roche, catalog no. 04693116001, Mannheim, Germany). The lysates were incubated on ice for 40 min and then centrifuged at 12,000× *g* for 15 min at 4 °C. Protein concentrations were determined using the BCA protein assay kit (Thermo Fisher Scientific, Rockford, IL, USA).

Immunoprecipitation was performed using the Dynabeads™ Protein G Immunoprecipitation Kit (Thermo Fisher Scientific, Waltham, MA, USA). Magnetic beads were incubated with 1.5 μL of GRP78 antibody in 100 μL of antibody binding and washing buffer for 15 min at room temperature with rotation. The bead–antibody complexes were resuspended in lysis buffer and incubated overnight at 4 °C to allow binding of the GRP78 antigen. The bead–antibody–antigen complexes were washed three times with 200 μL washing buffer, then resuspended in 30 μL lysis buffer mixed with 5 μL of 6× sample buffer and denatured at 95 °C for 5 min. The samples were loaded onto a 10% SDS-PAGE gel for protein separation.

Following electrophoresis, proteins were transferred to a 0.45 μm nitrocellulose membrane. The membrane was blocked in 5% nonfat dry milk prepared in TBST (25 mM Tris-HCl, pH 7.4; 125 mM NaCl; 0.05% Tween 20) for 1 h and 30 min. It was then incubated overnight at 4 °C with the primary antibody against ubiquitin. The next day, the membrane was incubated for 1 h at room temperature with HRP-conjugated secondary anti-mouse IgG antibody diluted 1:2000 in 5% skim milk in TBST. Protein bands were visualized using enhanced chemiluminescence and quantified by densitometry with the iBright™ CL 1500 Imaging System (Invitrogen™, cat no. A44240, Carlsbad, CA, USA) and the molecular weight were compared against the marker (Precision Plus Protein Dual Color Standards^TM^, BioRad, cat no.1610374, Hercules, CA, USA). Band intensities were analyzed using ImageJ software (version 1.52a, National Institutes of Health, Bathesda, MD, USA).

### 2.11. Statistical Analysis

Results are presented as the mean ± standard deviation (SD) based on three or more independent biological replicates. Statistical analysis for multiple group comparisons was conducted using one-way ANOVA followed by a post hoc test with GraphPad Prism version 9.0 (GraphPad Software, La Jolla, CA, USA). Differences were considered statistically significant at *p*-values below 0.05.

#### Statistical Analysis—DepMap

Statistical comparisons between groups were performed using a two-sided Mann–Whitney U test implemented in the SciPy library. This non-parametric test was selected due to the non-normal and highly skewed distribution of dependency scores, which are bounded between 0 and 1 and exhibit a pronounced ceiling effect. A *p*-value < 0.05 was considered statistically significant.

### 2.12. Molecular Docking

The X-ray crystal structure of human GRP78 ATPase domain (PDB ID: 3LDP [13]) was downloaded from the RCSB Protein Data Bank (http://www.rcsb.org/). Prior to docking simulation, all solvent molecules and the co-crystallized ligand were removed to prepare the protein structure. The SMILES representations of the ligands were obtained from the PubChem database (http://pubchem.ncbi.nlm.nih.gov/) and converted into 3D PDB format using the Online SMILES Translator and Structure File Generator (http://cactus.nci.nih.gov/translate/, accessed on 15 March 2025). PDBQT file formats for GRP78 protein and the ligands were generated using AutoDockFR 1.0 [14]. Molecular docking was carried out using AutoDock Vina version 1.2.7 [15]. The docking grid was centered on the coordinates of the original co-crystallized ligand within the active site, using a grid box size of 20 × 20 × 20 Å and center coordinates x = 17.52, y = −9.15, and z = 5.35. The best docking pose with the lowest AutoDock Vina score of the GRP78–Cypripedin complex was selected for further analysis. 3D visualization was performed using ChimeraX 1.10 [16], and 2D interaction diagram was analyzed using Discovery Studio Visualizer 2024 (BIOVIA, San Diego, CA, USA).

### 2.13. DepMap

#### 2.13.1. DepMap Data Acquisition and Preprocessing

Genome-wide CRISPR gene dependency data were obtained from the Cancer Dependency Map (DepMap Public release 25Q3). Gene-level dependency probabilities were downloaded as the CRISPRGeneDependency.csv dataset. Cell line annotations, including lineage classification and standardized cell line names, were retrieved from the corresponding Model.csv file.

The dependency score for HSPA5 (GRP78) was extracted using the annotated gene identifier (“HSPA5 (3309)”). Cell line identifiers in the dependency matrix (ModelID) were matched to annotation data via an inner merge to obtain lineage information and standardized cell line names. Data preprocessing and integration were performed using Python (version 3.11.3; Clang 14.0.6) with the pandas library.

#### 2.13.2. Lineage Grouping and Data Stratification

Cell lines were categorized based on the “OncotreeLineage” annotation. For comparative analysis, cell lines were grouped into lymphoid and non-lymphoid (“Other”) categories. The lymphoid group included hematopoietic malignancies of lymphoid origin, while all remaining lineages were classified as “Other”.

#### 2.13.3. Data Visualization

Dependency distributions were visualized using boxplots with overlaid individual data points (strip plots) generated using the seaborn and matplotlib libraries. Boxplots represent the interquartile range (IQR), with median values indicated by central lines and whiskers extending to 1.5 × IQR. Individual cell lines are displayed as semi-transparent points to illustrate data distribution and outliers.

## 3. Results

### 3.1. Cytotoxicity and IC_50_ Determination of Phenanthrene-Type Compounds in Jurkat Cells

**Figure 1:**
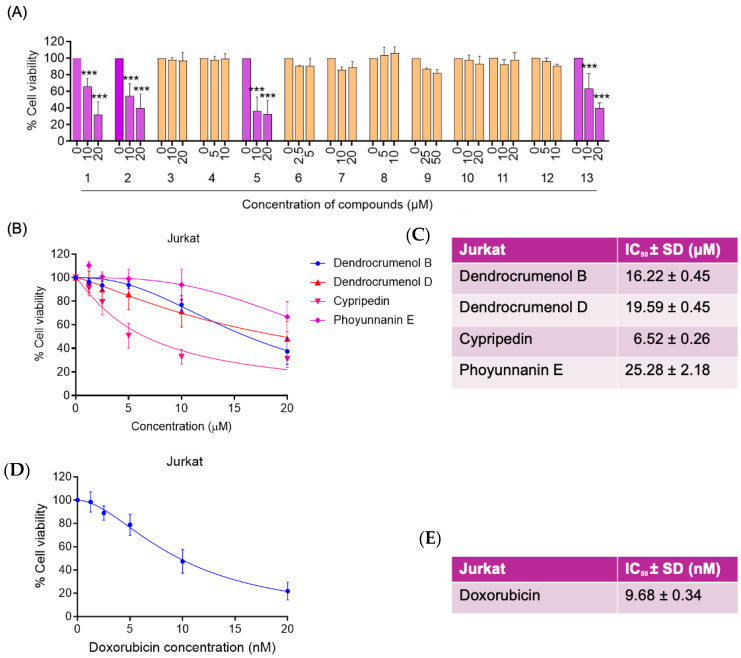
Screening of natural products derived phenanthrene-type compounds for cytotoxicity in Jurkat T-ALL cells and IC_50_ determination for lead hits. (**A**) Jurkat cells were treated for 24 h with 13 natural compounds at the indicated concentrations (µM), and cell viability was measured by WST-1 assay. List of 13 compounds are (1) Dendrocrumenol B (2) Dendrocrumenol D (3) Gigantol (4) 3,7-dihydroxy-2,4,6-trimethoxyphenanthrene (5) Cypripedin (6) Moscatilin (7) Crepidatin (8) Dendrocandin B (9) Syringaresinol (10) Dendroflorin (11) Tetracosyl (E)-p-coumarate (12) Dendrofalconerol B (13) Phoyunnanin E. Bars represent mean ± SD (n = 4). Compounds reducing viability by ≥30% at 10 or 20 µM are shown in purple; all others in orange. Statistical significance versus vehicle (0 µM) is indicated: (*** *p* < 0.001 vs. control; one-way ANOVA with Tukey’s post hoc test). (**B**) Dose–response curves for Dendrocrumenols B and D, Cypripedin, Phoyunnanin E. Data are mean ± SD (n = 3) and were fit by nonlinear regression to calculate IC_50_ values. (**C**) Table of IC_50_ values derived from the curves in (**B**). Cypripedin showed the lowest IC_50_ among natural compounds, identifying it as the lead for further mechanistic studies. (**D**) Dose–response curves for the positive control doxorubicin (0.01–20 nM). Data are mean ± SD (n = 3) and were fit by nonlinear regression to calculate IC_50_ values. (**E**) Table of IC_50_ values derived from the curves in (**D**).

We first screened thirteen natural compounds (1) Dendrocrumenol B (2) Dendrocrumenol D (3) Gigantol (4) 3,7-dihydroxy-2,4,6-trimethoxyphenanthrene (5) Cypripedin (6) Moscatilin (7) Crepidatin (8) Dendrocandin B (9) Syringaresinol (10) Dendroflorin (11) Tetracosyl (E)-p-coumarate (12) Dendrofalconerol B (13) Phoyunnanin E) (Chemical structure of all compounds were shown in Supplementary Figure S1) at the two maximum doses without observed precipitations, for 24 h in Jurkat cells via WST-1 assay. Most agents exhibited minimal cytotoxicity (viability ≥ 90%), whereas Cypripedin induced a pronounced, dose-dependent loss of viability (38 ± 5% at 10 µM; 37 ± 4% at 20 µM; *** *p* < 0.001). To quantify potency, we generated full dose–response curves for Dendrocrumenol B, Dendrocrumenol D, Cypripedin, Phoyunnanin E and the positive control doxorubicin (0.01–20 nM). Nonlinear regression yielded IC_50_ values of 16.22 µM for Dendrocrumenol B, 19.59 µM for Dendrocrumenol D, 6.52 µM for Cypripedin, 25.28 µM for Phoyunnanin E, and 9.68 nM for doxorubicin (Figure 1). Cypripedin’s low-micromolar IC_50_ further underscores its promise as a lead cytotoxic agent for T-cell malignancies. To further evaluate the generalizability of this effect, cypripedin was tested in an additional T-ALL cell line (MOLT-4), where it exhibited a lower IC_50_ value of 2.72 µM, indicating higher sensitivity compared to Jurkat cells (Supplementary Figure S2).

### 3.2. Induction of Apoptosis by Cypripedin

**Figure 2:**
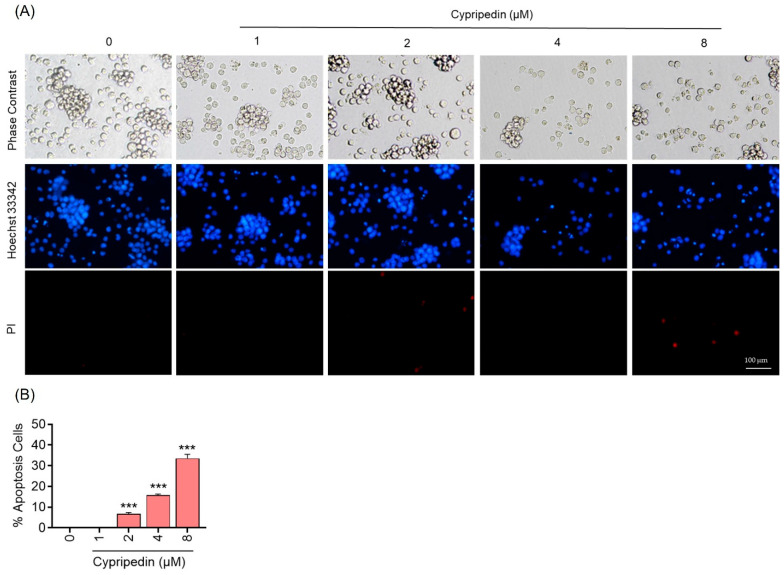

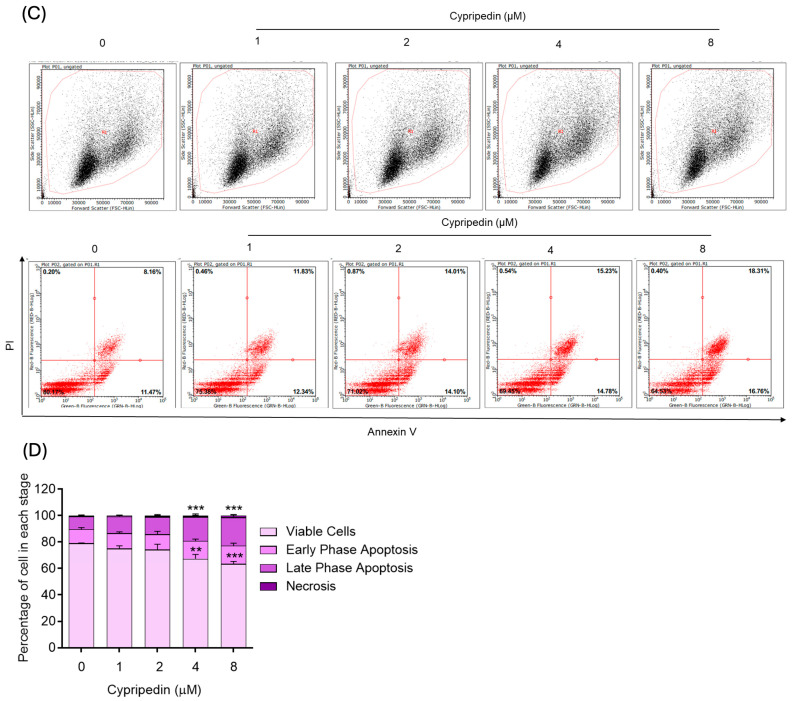
Cypripedin induces dose-dependent apoptosis in Jurkat T-ALL cells. (**A**) Jurkat cells were treated for 24 h with 0, 1, 2, 4, or 8 µM Cypripedin and then stained with Hoechst 33342 (blue) and propidium iodide (PI; red) to distinguish apoptotic versus necrotic cells. Representative phase-contrast images show cell shrinkage and membrane blebbing at increasing Cypripedin concentrations. Corresponding fluorescence micrographs of Hoechst 33342-stained nuclei reveal chromatin condensation and fragmentation; PI staining indicates minimal necrosis. (**B**) Quantification of apoptotic cells based on nuclear morphology (condensation/fragmentation) and PI exclusion. Bars represent mean ± SD (n = 3); (*** *p* < 0.001 vs. control; one-way ANOVA with Tukey’s post hoc test). Scale bar = 100 µm (magnification: 20×). (**C**) Jurkat cells were treated for 24 h with 0, 1, 2, 4, or 8 µM Cypripedin and stained with Annexin V−FITC and propidium iodide (PI). Top panels: Representative density plots showing total cell populations gated to exclude debris. Bottom panels: Corresponding Annexin V/PI dot plots. Quadrants denote viable (Annexin V−/PI−, (**lower left**)), early apoptotic (Annexin V+/PI−, (**lower right**)), late apoptotic or secondary necrotic (Annexin V+/PI+, (**upper right**)), and necrotic (Annexin V−/PI+, (**upper left**)) cells. (**D**) Percentages of each population are indicated within the quadrants. With increasing Cypripedin concentration, the proportions of early and late apoptotic cells rise markedly, while necrosis remains minimal. Cells (n = 3) were analyzed using Flowjo; plots are representative of three independent experiments (** *p* < 0.01 vs. control, *** *p* < 0.001 vs. control; one-way ANOVA with Tukey’s post hoc test).

To investigate whether the cytotoxicity induced by Cypripedin reflects apoptotic cell death, Jurkat cells were treated with increasing concentrations of Cypripedin (0, 1, 2, 4, 8 µM) for 24 h and stained with Hoechst 33342 and propidium iodide (PI). Phase-contrast images revealed dose-dependent morphological changes characteristic of apoptosis, including cell shrinkage and membrane blebbing. Hoechst-stained nuclei showed chromatin condensation and fragmentation, while PI uptake remained minimal, indicating low levels of necrosis (Figure 2A). Quantification of apoptotic cells based on PI exclusion and nuclear morphology demonstrated a significant increase in apoptotic cells from 0 ± 2% in controls to 38 ± 4% at 8 µM (*** *p* < 0.001 vs. control) (Figure 2B). These findings confirm that Cypripedin primarily induces apoptosis in Jurkat cells.

To corroborate the Hoechst/PI findings, we performed Annexin V–FITC/PI staining followed by flow cytometry on Jurkat cells treated with Cypripedin (0, 1, 2, 4, 8 µM) for 24 h. Representative dot plots (Figure 2C) show four quadrants: viable (Annexin V−/PI−), early apoptotic (Annexin V+/PI−), late apoptotic or secondary necrotic (Annexin V+/PI+), and necrotic (Annexin V−/PI+). Quantification of apoptotic populations (sum of early and late fractions) revealed a dose-dependent increase when comparing from control and Cypripedin treatment in dose dependent fashion (*** *p* < 0.001) (Figure 2C,D). Early apoptosis predominated at lower concentrations, whereas higher doses led to an elevated proportion of late apoptotic cells. These flow cytometry data further confirm that Cypripedin induces apoptosis in Jurkat cells in a concentration-dependent manner. Consistently, in MOLT-4 cells, cypripedin induced significant increases in both early and late apoptotic populations, following a dose-dependent trend as observed in Jurkat cells (Supplementary Figure S3).

### 3.3. Synergistic Cytotoxicity of Cypripedin with Bortezomib

**Figure 3:**
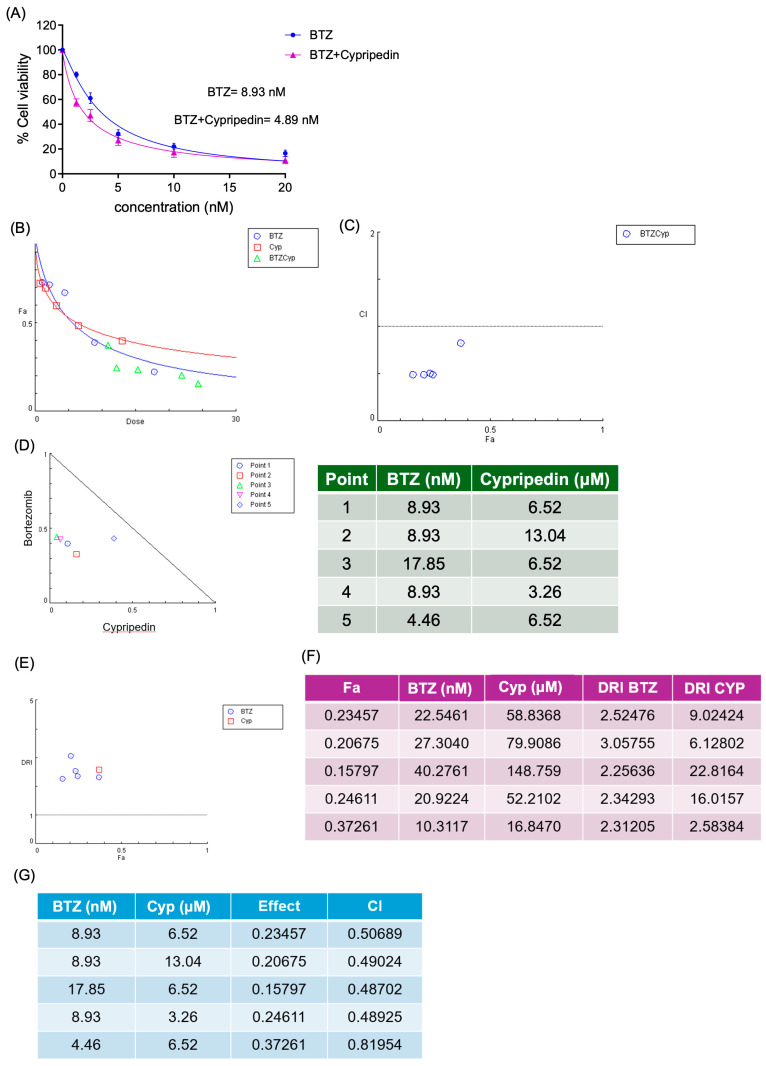
Synergy analysis of Cypripedin and bortezomib (BTZ) in Jurkat T-ALL cells. (**A**) Jurkat cells were treated with pretreatment 1h for Cypripedin (4 μM) follow by BTZ with various concentration of (0–20 nM) for 24 h and measure by WST-1 assay. Dose–response curves were fitted using a four-parameter logistic (4PL) nonlinear regression model. The calculated IC_50_ values were 8.93 nM for BTZ alone and 4.89 nM for BTZ in combination with Cypripedin. IC_50_ is defined as the concentration corresponding to the midpoint between the upper (Top) and lower (Bottom) plateaus of the fitted curve. (**B**) CompuSyn analysis was performed on cytotoxicity data to assess the synergistic interaction between BTZ and Cypripedin in Jurkat cells. Dose–response curves for BTZ, Cypripedin, and their combination were generated. (**C**) Combination Index (CI) plots showed CI values less than 1, indicating a synergistic effect between BTZ and Cypripedin. (**D**) Isobolograms illustrated the effective doses required to achieve 50% (Fa 0.5) inhibition for each drug alone, with synergism indicated when dose combinations fell below the corresponding Fa lines. (**E**) Dose Reduction Index (DRI) values for the BTZ and Cypripedin combination are shown, where DRI values greater than 1 suggest a beneficial interaction. (**F**,**G**) The presented data were generated using CompuSyn and are based on three independent experiments. Fa, fraction affected.

Given the clinical relevance of bortezomib (BTZ) in T-ALL treatment, we evaluated the combination of Cypripedin and BTZ for synergistic effects in Jurkat cells. Cells were treated for 24 h across a fixed-ratio dose matrix, and cell viability was assessed by WST-1. Combination index (CI) values were calculated according to the Chou–Talalay method from three independent trials (Figure 3B–F). In all trials, the CI values less than 1, indicating a synergistic effect between BTZ and Cypripedin. The summary plot confirmed synergy across multiple effect levels. These data demonstrate that Cypripedin and BTZ act synergistically to enhance cytotoxicity in T-ALL cells, supporting combined therapeutic potential.

To quantify the enhancement of BTZ efficacy by Cypripedin, we compared dose–response curves for BTZ alone versus BTZ combined with a fixed at 4 µM Cypripedin. Jurkat cells were treated for 24 h with BTZ (0–20 nM) in the presence or absence of Cypripedin, and viability was assessed by WST-1. Nonlinear regression analysis revealed an IC_50_ of 8.93 nM for BTZ alone and the reduction of IC_50_ to 4.89 nM for the BTZ + Cypripedin combination (Figure 3A). These data indicate that Cypripedin potentiates BTZ cytotoxicity by nearly two-fold, supporting the synergistic interaction observed in the combination index analysis. Consistently, similar synergistic effects were observed in MOLT-4 cells, where cypripedin enhanced the cytotoxicity of bortezomib under comparable conditions (Supplementary Figure S4) (Raw data is available in Supplementary Figure S7).

### 3.4. Induction of Endoplasmic Reticulum Stress Pathways by Cypripedin

**Figure 4:**
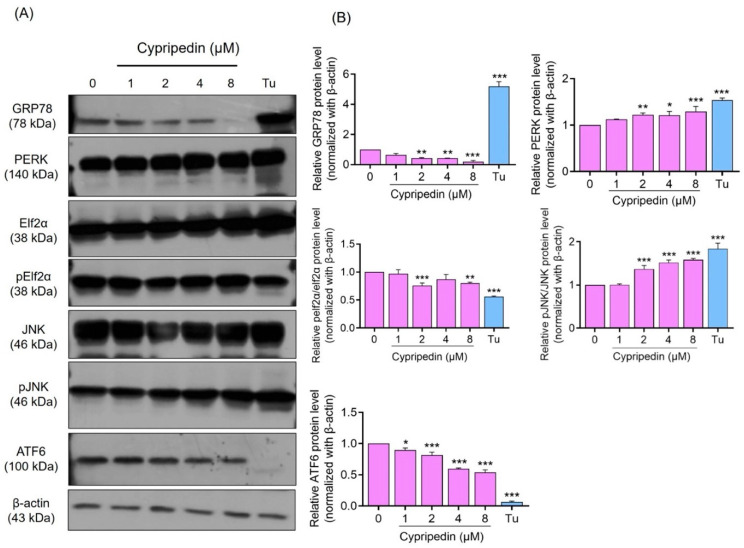
Cypripedin activates unfolded protein response signaling in Jurkat T-ALL cells. Jurkat cells were treated for 24 h with vehicle (0 µM), 1, 2, 4, or 8 µM Cypripedin, or 2.5 µM tunicamycin (Tu) as a positive control, and lysates were analyzed by Western blot. β-Actin serves as a loading control. Representative blots (**A**) show dose-dependent modulation in GRP78, phosphorylated PERK (p-PERK), phosphorylated eIF2α (p-eIF2α), phosphorylated JNK (p-JNK), and full-length ATF6. (**B**) Relative protein expression normalized with β-Actin, compared with control (* *p* < 0.05 vs. control ** *p* < 0.01 vs. control, *** *p* < 0.001 vs. control; one-way ANOVA with Tukey’s post hoc test).

To evaluate the effect of Cypripedin on ER stress signaling, we examined the expression of major unfolded protein response (UPR) markers by Western blot analysis. As shown in Figure 4, Cypripedin treatment resulted in a modest change in phosphorylation of PERK and its downstream effector eIF2α, indicating minimal activation of the PERK–eIF2α axis. In contrast, phosphorylation of JNK was robustly elevated in a dose-dependent manner, consistent with activation of pro-apoptotic ER stress signaling. The expression of GRP78, a key ER chaperone and stress sensor, was significantly reduced across all concentrations of Cypripedin, unlike the strong induction observed with tunicamycin. Furthermore, full-length ATF6 protein levels were markedly decreased following Cypripedin treatment. This reduction may reflect either suppressed ATF6 expression or enhanced proteolytic processing into its active cleaved form (ATF6f), which translocates to the nucleus to mediate target gene expression. Collectively, these data suggest that Cypripedin preferentially activates the JNK-mediated apoptotic arm of the UPR while suppressing or altering adaptive pathways, including GRP78 and ATF6 signaling. Consistently, in MOLT-4 cells, Cypripedin also reduced GRP78 expression, supporting a similar effect on ER chaperone regulation in an additional T-ALL model (Supplementary Figure S5).

To further contextualize the relevance of GRP78 in hematologic malignancies, we analyzed CRISPR-based gene dependency data from the DepMap dataset. HSPA5 (GRP78) exhibited consistently high dependency scores across cancer cell lines, reflecting its central role in ER proteostasis. Notably, a modest but statistically significant increase in dependency was observed in lymphoid compared to non-lymphoid lineages (Mann–Whitney U test, *p* = 7.68 × 10^−4^). Given that T-ALL originates from malignant T-lineage lymphoid cells, this lineage-specific dependency supports the potential vulnerability of these cells to perturbation of GRP78 function. In line with this, our experimental findings demonstrate that cypripedin reduces GRP78 expression and induces cytotoxicity in T-cell leukemia models, including Jurkat and MOLT-4 cells, suggesting that disruption of ER proteostasis may represent a therapeutically exploitable mechanism in T-cell malignancies (Supplementary Figure S6).

### 3.5. GRP78 Downregulation Involved Both Transcriptional and Ubiquitin–Proteasomal Regulation

**Figure 5:**
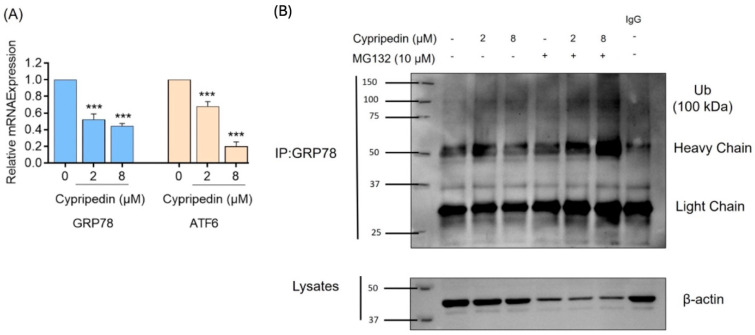
Cypripedin downregulates UPR gene transcripts and promotes ubiquitination and proteasomal turnover of GRP78 in Jurkat T-ALL cells. (**A**) Cells were treated for 24 h with vehicle (0 µM), 2, or 8 µM Cypripedin, and total RNA was analyzed by SYBR Green qRT-PCR. Bar graphs show relative mRNA expression of GRP78 (left, blue bars) and ATF6 (right, orange bars) normalized to GAPDH and plotted relative to control (mean ± SD, n = 3). (*** *p* < 0.001 vs. control; one-way ANOVA with Tukey’s post hoc test). (**B**) Jurkat cells were treated for 24 h with vehicle (Ctrl) or Cypripedin (2 or 8 µM) in the absence or presence of the proteasome inhibitor MG132 (10 µM). Input lysates. Western blots of total cell lysates probed for β-actin (loading control). Immunoprecipitation (IP) and ubiquitin blot. Endogenous GRP78 was immunoprecipitated from the same lysates and blotted with anti-ubiquitin antibody. Smearing and laddering of high-molecular-weight bands suggest that Cypripedin is associated with increased GRP78 ubiquitination, which is further enhanced by MG132. IgG lanes serve as negative IP controls.

To elucidate the mechanism underlying the observed decrease in GRP78 protein, we measured mRNA levels of GRP78 and ATF6 by real-time RT-PCR. Jurkat cells were treated with 0, 2 and 8µM cypripedin for 24 h. As shown in Figure 5A, GRP78 transcript levels were significantly downregulated at both 2 and 8 µM compared to the control (*** *p* < 0.001). Similarly, ATF6 mRNA expression was also significantly decreased at 2 and 8 µM, with a more pronounced reduction observed at 8 µM (*** *p* < 0.001). These data indicate that Cypripedin selectively suppresses UPR gene transcription, offering a potential explanation for its impact on ER chaperone abundance and stress signaling. Similarly, comparable reductions in GRP78 mRNA levels were observed in MOLT-4 cells following cypripedin treatment under comparable conditions (Supplementary Figure S5).

To confirm whether Cypripedin promotes GRP78 degradation via the ubiquitin–proteasome pathway, we immunoprecipitated endogenous GRP78 from Jurkat cell lysates treated with Cypripedin (0, 2, 8 µM) ± 10 µM MG132 for 24 h. Western blotting of GRP78 IPs with ubiquitin antibody revealed a trend toward increased ubiquitinated GRP78 species, which was more apparent upon MG132 co-treatment (Figure 5B). Input controls verified equal β-actin levels (Figure 5B). These data suggest that Cypripedin is associated with increased GRP78 ubiquitination, providing a potential mechanistic link for its suppression of ER chaperone levels.

### 3.6. In Silico Docking of Cypripedin to the GRP78 ATPase Domain

**Figure 6:**
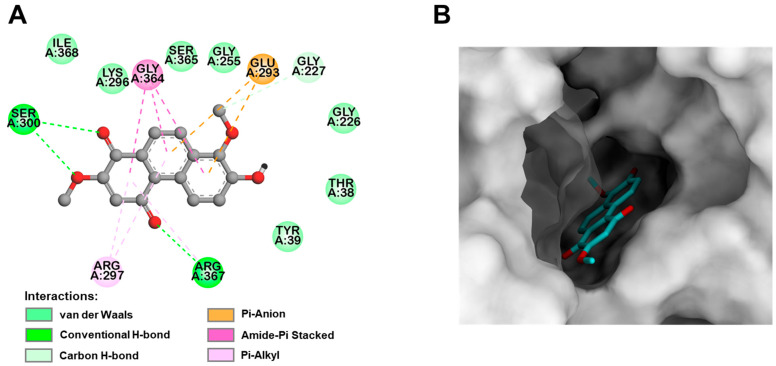
In silico docking of Cypripedin to the GRP78 ATPase domain. (**A**) Binding interactions of Cypripedin with the GRP78 ATPase domain were illustrated by 2D interaction diagram and (**B**) a 3D binding mode representation, as obtained from molecular docking analysis.

To assess the binding mode and affinity of Cypripedin toward the GRP78 ATPase domain, molecular docking was performed using AutoDock Vina. Cypripedin was docked against the ATPase domain crystal structure alongside known inhibitors, including an adenosine-derived compound (Vina score: −9.175 kcal/mol), EGCG (−8.913 kcal/mol), and isoliquiritigenin (−8.103 kcal/mol). Cypripedin yielded a docking score of −7.630 kcal/mol, suggesting a favorable, though slightly lower, binding affinity compared to the reference inhibitors. Visualization of the top-ranked binding pose (Figure 6) showed that Cypripedin occupies the ATP binding site of GRP78 and forms hydrogen bonds with key residues Ser300 and Arg367. Additionally, Pi-related interactions, including Pi-anion, amide–Pi stacking, and Pi–alkyl interactions were observed with the residues Glu293, Arg297, Gly364, and Arg367.

## 4. Discussion

Our study identifies Cypripedin as a potent inducer of apoptosis in Jurkat T-ALL cells, demonstrating an IC_50_ of 6.52 µM and robust activation of intrinsic apoptotic pathways. The compound’s ability to synergize with bortezomib—evidenced by combination index values below 0.5 and 1.8-fold reduction in the proteasome inhibitor IC_50_—suggests a promising strategy to enhance therapeutic efficacy while potentially lowering the required dose and mitigating systemic toxicity. Apoptosis induction by Cypripedin was confirmed through complementary assays. Hoechst 33342/PI staining provided morphological evidence of chromatin condensation, while Annexin V/PI flow cytometry quantified both early and late apoptotic populations. These multimodal data validate Cypripedin’s pro-apoptotic activity and are consistent with prior reports of phenanthrene derivatives inducing cytotoxicity via mitochondrial pathways in solid tumor models [17].

Mechanistically, Western blot analysis revealed activation of multiple branches of the unfolded protein response (UPR), including the alteration of GRP78, phosphorylated PERK and eIF2α, ATF6 and upregulation of p-JNK. This broad engagement of ER stress signaling distinguishes Cypripedin from many natural products, which typically target individual UPR nodes. Notably, at higher concentrations, Cypripedin selectively downregulated GRP78 and ATF6 transcripts, suggesting a shift from adaptive to terminal UPR—a transition aligned with ER stress-mediated apoptosis mechanisms described by Hetz [18].

A key novel finding of this study is the promotion of GRP78 ubiquitination by Cypripedin, demonstrated by immunoprecipitation assays and enhanced by proteasome blockade with MG132. GRP78, a central chaperone that maintains ER protein-folding homeostasis, is frequently upregulated in hematologic malignancies and has been implicated in chemoresistance [19]. By promoting GRP78 turnover, Cypripedin compromises the cell’s ability to manage ER stress, thereby sensitizing cells to proteasome inhibition. Molecular docking analysis supports this proposed mechanism, predicting that Cypripedin binds within the ATPase cleft of GRP78, forming hydrogen bonds with Ser300 and Arg367, and engaging surrounding hydrophobic residues. This interaction is likely to destabilize the chaperone’s conformation, potentially exposing lysine residues for recognition by E3 ubiquitin ligases and facilitating ubiquitination. Future studies should include site-directed mutagenesis of these contact residues, along with biophysical assays such as thermal shift and surface plasmon resonance, to validate direct binding.

Comparatively, other group have reported on natural products that modulate ER stress—for example, 7,8-dihydroxyflavone suppressing GRP78 in metabolic disease models and icariin inhibiting the IRE1α–XBP1 pathway in neurons [20,21]. However, few studies have demonstrated both induction of ER stress and targeted degradation of a key UPR chaperone. Cypripedin’s dual functionality—activating stress responses while promoting chaperone depletion—introduces a novel paradigm in natural product pharmacology. Therapeutically, the observed synergy with bortezomib highlights the potential for combination regimens in T-ALL and possibly other malignancies that rely on proteostasis. The ability to lower effective doses of proteasome inhibitors may reduce associated toxicities, such as peripheral neuropathy and myelosuppression, which currently limit clinical use. Furthermore, targeting GRP78 may help overcome resistance mechanisms not addressed by existing therapies. While our in vitro data are comprehensive, in vivo validation is critical. Studies on pharmacokinetics, bioavailability, and toxicity are required to assess translational potential. Additionally, identifying the specific E3 ligases responsible for GRP78 ubiquitination could provide mechanistic insight and identify new therapeutic targets. Structure–activity relationship (SAR) studies of Cypripedin and its analogs may further enhance its binding affinity and selectivity.

In conclusion, Cypripedin is a multifaceted natural compound that induces ER stress-mediated apoptosis and potentiates the cytotoxic effects of proteasome inhibition, with associated GRP78 ubiquitination. This integrated mechanism of action represents a novel therapeutic avenue for T-ALL and other proteostasis-dependent cancers, meriting further preclinical investigation, particularly in animal models.

## 5. Conclusions

Cypripedin is a promising natural compound that induces apoptotic death, enhances proteasome inhibitor efficacy, and targets ER stress pathways associating GRP78 ubiquitination in Jurkat T-ALL cells. These data support its development as a novel combination therapy for T-ALL.

## Data Availability

The raw datasets generated and analyzed during the current study, including source data for all quantitative graphs, uncropped Western blot images, and fluorescence microscopy images from cell staining experiments, are available in the Supplementary Information files associated with this article. The authors confirm that the data supporting the findings of this study are available within the article and its Supplementary Materials.

## Supplementary Materials

The following supporting information can be downloaded at: https://www.mdpi.com/article/10.3390/molecules31111823/s1, Figure S1. Chemical structure of natural products derived phenanthrene-type compounds., Figure S2. Natural products derived phenanthrene-type compounds for cytotoxicity in MOLT4 cells and IC_50_ determination., Figure S3. Cypripedin induces dose-dependent apoptosis in MOLT4 cells., Figure S4. Synergy analysis of Cypripedin and bortezomib (BTZ) in MOLT4 cells., Figure S5. Cypripedin modulates GRP78 at transcriptional and translational level in MOLT4 cells., Figure S6. HSPA5 (GRP78) dependency across cancer cell lineages., Figure S7. Raw data for Figure 3A–G and CompuSyn Report., Figure S8. Overlayed Blots, whole membrane.
