# Supplementary material for: Cypripedin Induces Apoptosis and Synergizes with Bortezomib via ER Stress Mediated Ubiquitination of GRP78 in T-Cell Acute Lymphoblastic Leukemia
Source: Molecules. 2026 May 25;31(11):1823. doi: 10.3390/molecules31111823 (PMC13257771; doi:10.3390/molecules31111823)
Supplement: Supplementary file 1 [file molecules-31-01823-s001.zip › Supplementary Figures S1-S6.pptx]

## Slide 1
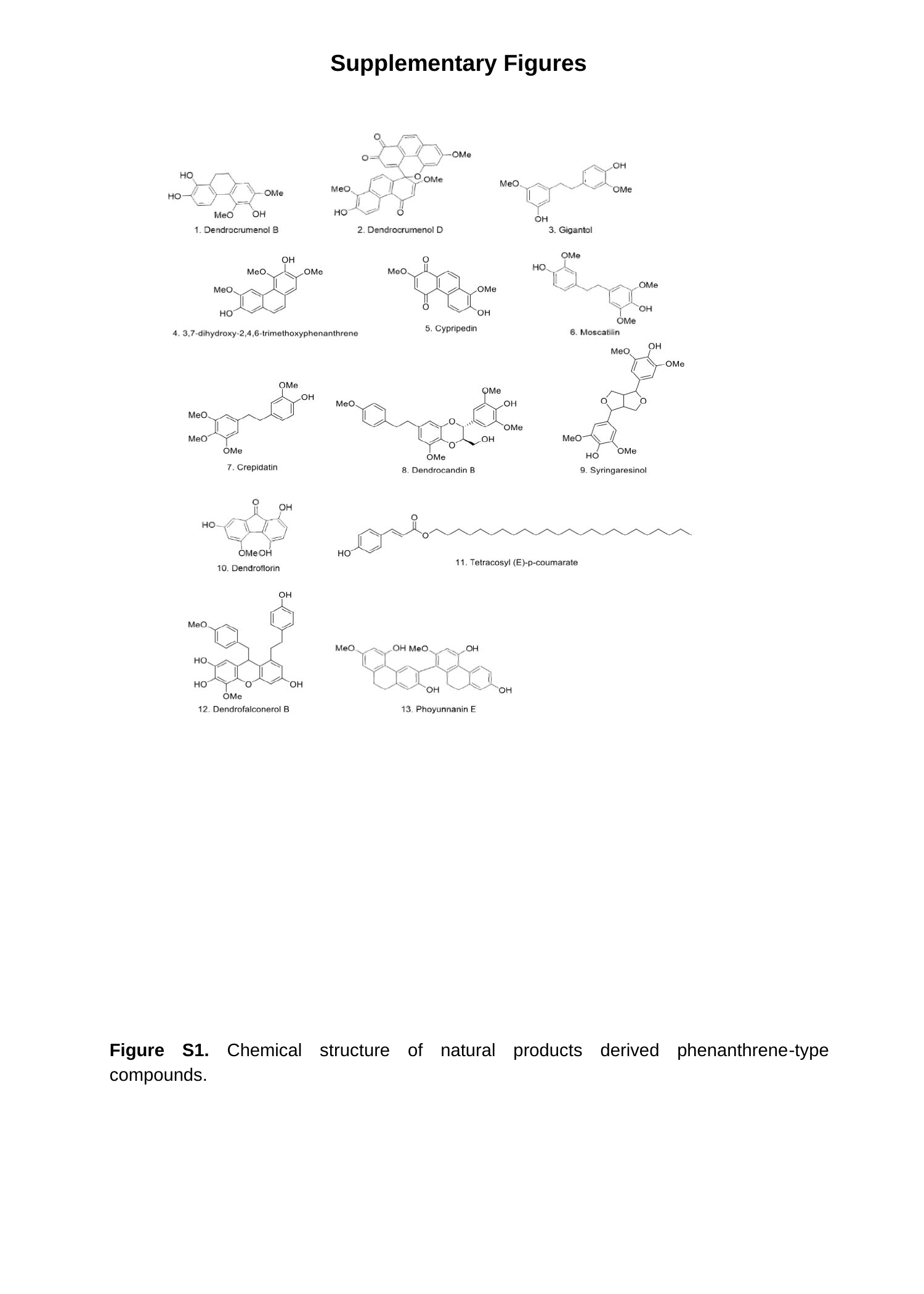

Supplementary Figures
Figure S1. Chemical structure of natural products derived phenanthrene‐type compounds.

## Slide 2
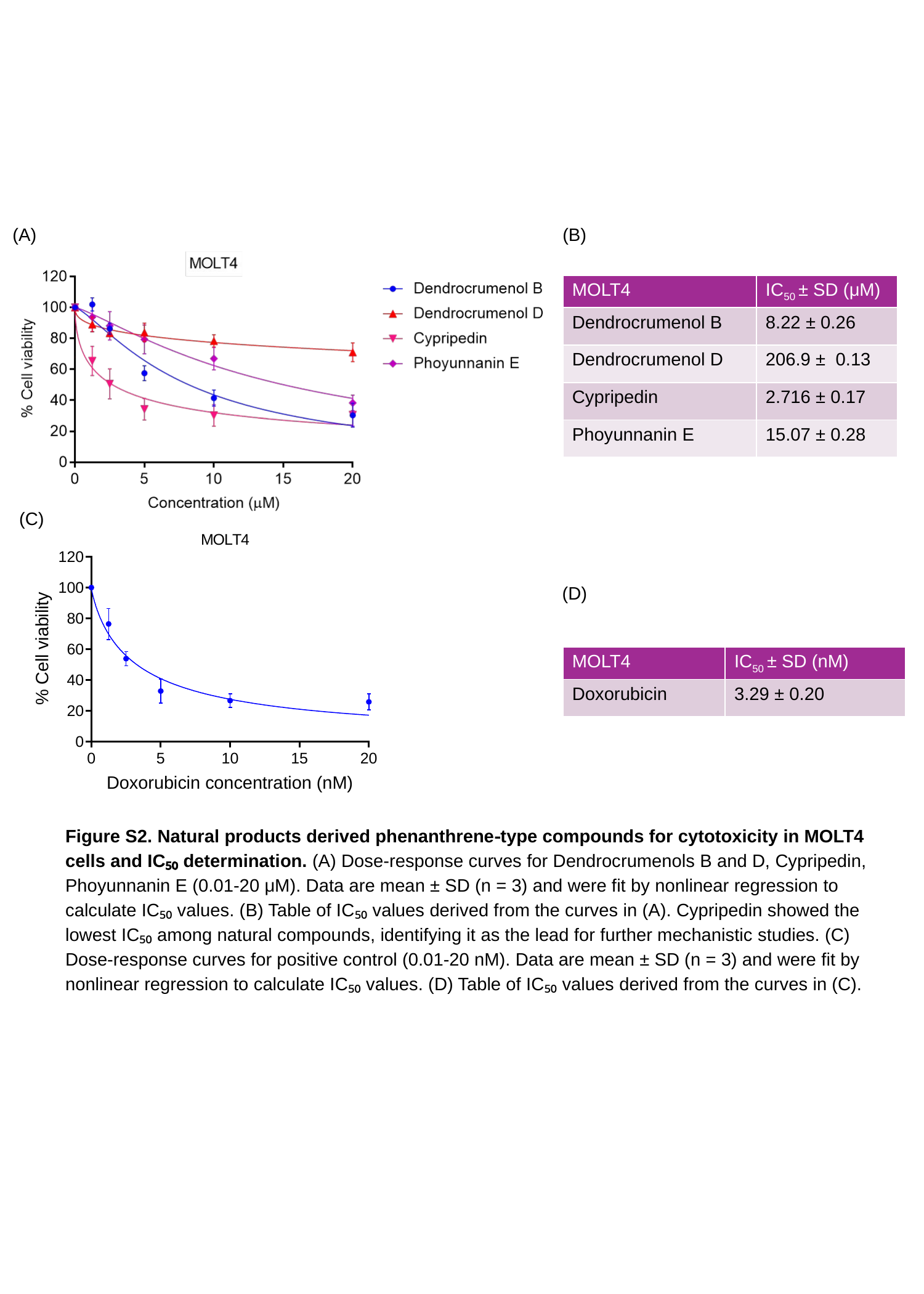

(A)
(B)
| MOLT4 | IC50 ± SD (μM) |
| --- | --- |
| Dendrocrumenol B | 8.22 ± 0.26 |
| Dendrocrumenol D | 206.9 ± 0.13 |
| Cypripedin | 2.716 ± 0.17 |
| Phoyunnanin E | 15.07 ± 0.28 |
(C)
(D)
| MOLT4 | IC50 ± SD (nM) |
| --- | --- |
| Doxorubicin | 3.29 ± 0.20 |
Figure S2. Natural products derived phenanthrene‐type compounds for cytotoxicity in MOLT4 cells and IC₅₀ determination. (A) Dose-response curves for Dendrocrumenols B and D, Cypripedin, Phoyunnanin E (0.01-20 μM). Data are mean ± SD (n = 3) and were fit by nonlinear regression to calculate IC₅₀ values. (B) Table of IC₅₀ values derived from the curves in (A). Cypripedin showed the lowest IC₅₀ among natural compounds, identifying it as the lead for further mechanistic studies. (C) Dose-response curves for positive control (0.01-20 nM). Data are mean ± SD (n = 3) and were fit by nonlinear regression to calculate IC₅₀ values. (D) Table of IC₅₀ values derived from the curves in (C).

## Slide 3
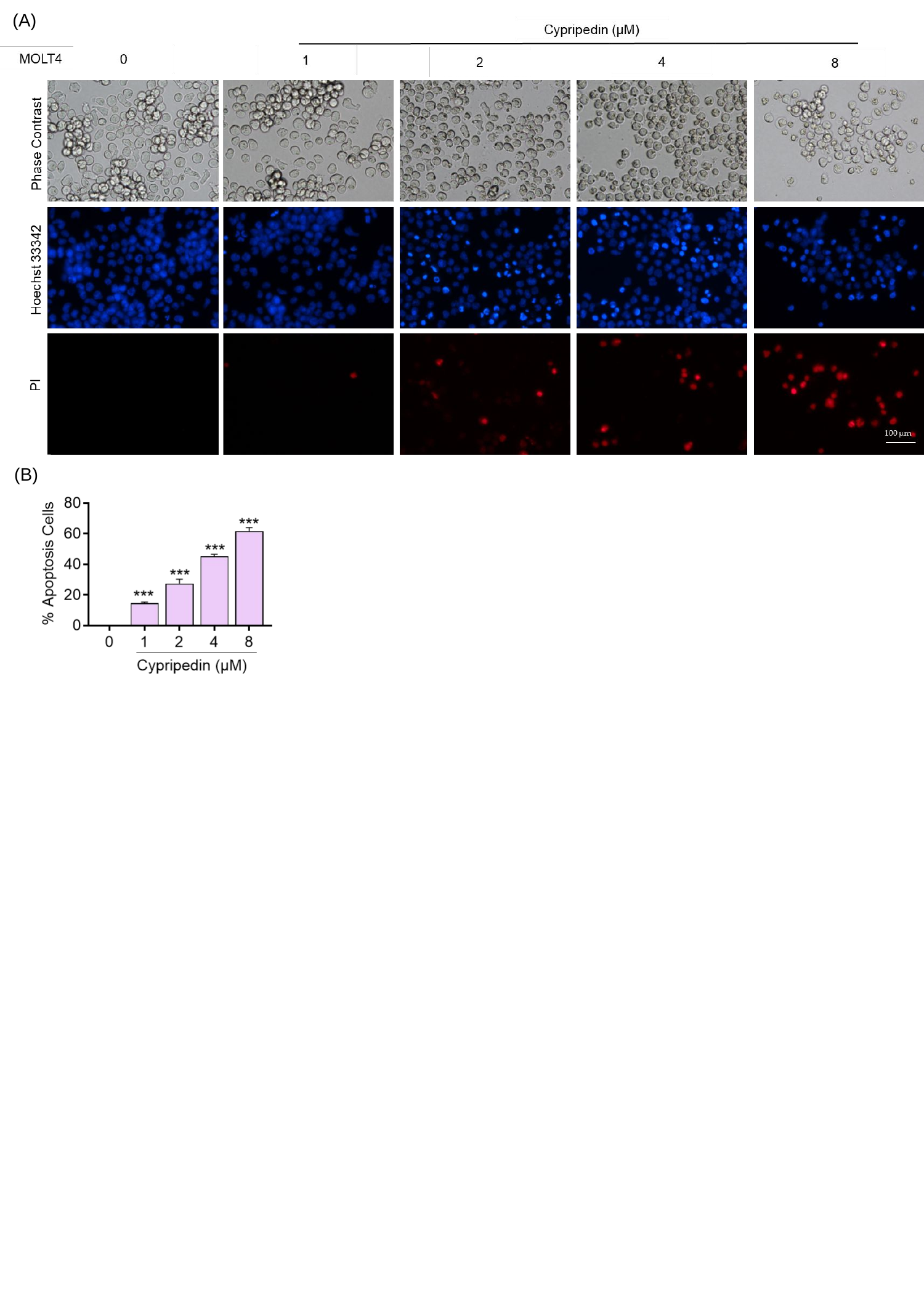

(A)
(B)

## Slide 4
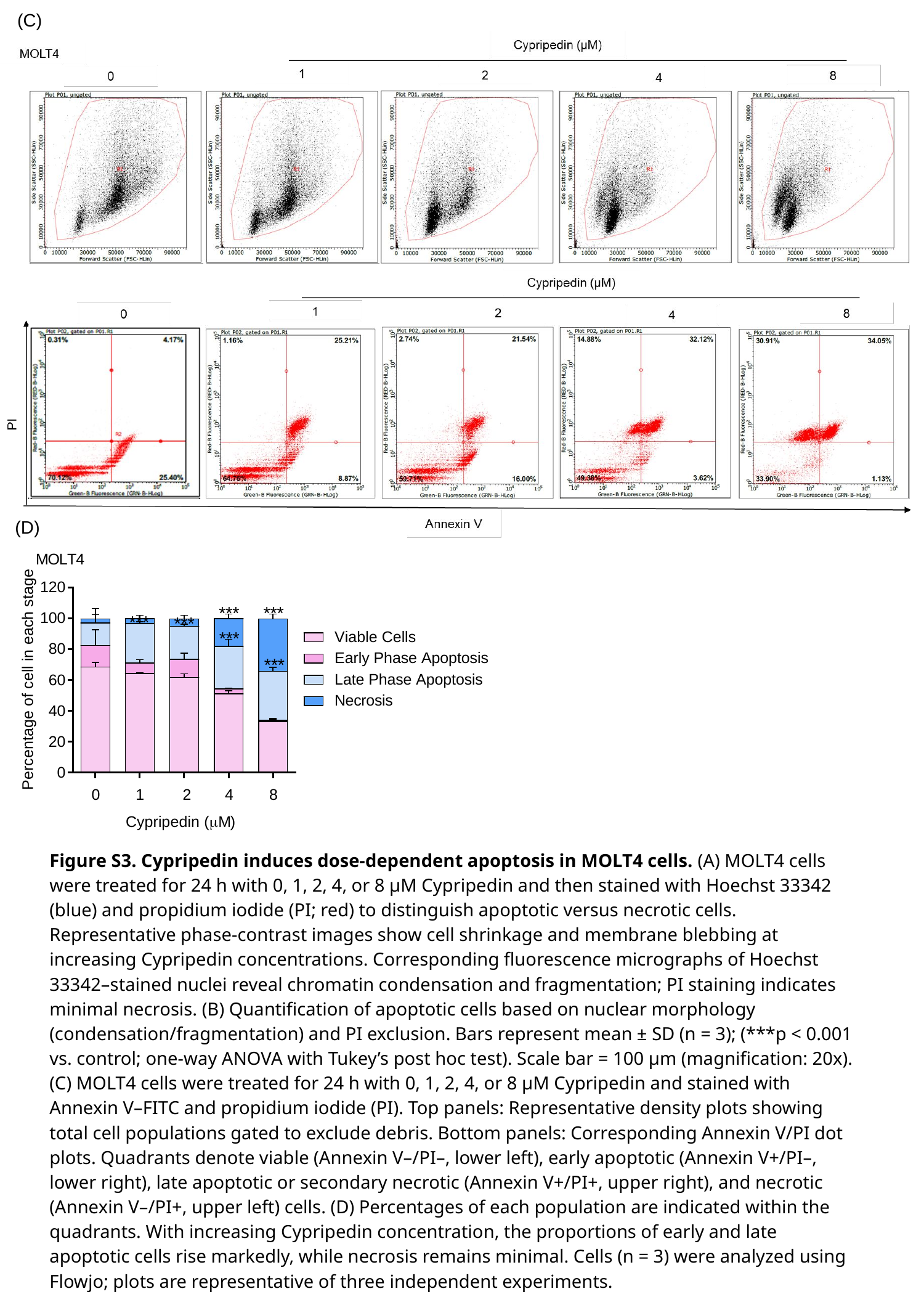

(C)
(D)
Figure S3. Cypripedin induces dose-dependent apoptosis in MOLT4 cells. (A) MOLT4 cells were treated for 24 h with 0, 1, 2, 4, or 8 μM Cypripedin and then stained with Hoechst 33342 (blue) and propidium iodide (PI; red) to distinguish apoptotic versus necrotic cells. Representative phase-contrast images show cell shrinkage and membrane blebbing at increasing Cypripedin concentrations. Corresponding fluorescence micrographs of Hoechst 33342–stained nuclei reveal chromatin condensation and fragmentation; PI staining indicates minimal necrosis. (B) Quantification of apoptotic cells based on nuclear morphology (condensation/fragmentation) and PI exclusion. Bars represent mean ± SD (n = 3); (***p < 0.001 vs. control; one-way ANOVA with Tukey’s post hoc test). Scale bar = 100 μm (magnification: 20x). (C) MOLT4 cells were treated for 24 h with 0, 1, 2, 4, or 8 μM Cypripedin and stained with Annexin V–FITC and propidium iodide (PI). Top panels: Representative density plots showing total cell populations gated to exclude debris. Bottom panels: Corresponding Annexin V/PI dot plots. Quadrants denote viable (Annexin V–/PI–, lower left), early apoptotic (Annexin V+/PI–, lower right), late apoptotic or secondary necrotic (Annexin V+/PI+, upper right), and necrotic (Annexin V–/PI+, upper left) cells. (D) Percentages of each population are indicated within the quadrants. With increasing Cypripedin concentration, the proportions of early and late apoptotic cells rise markedly, while necrosis remains minimal. Cells (n = 3) were analyzed using Flowjo; plots are representative of three independent experiments.

## Slide 5
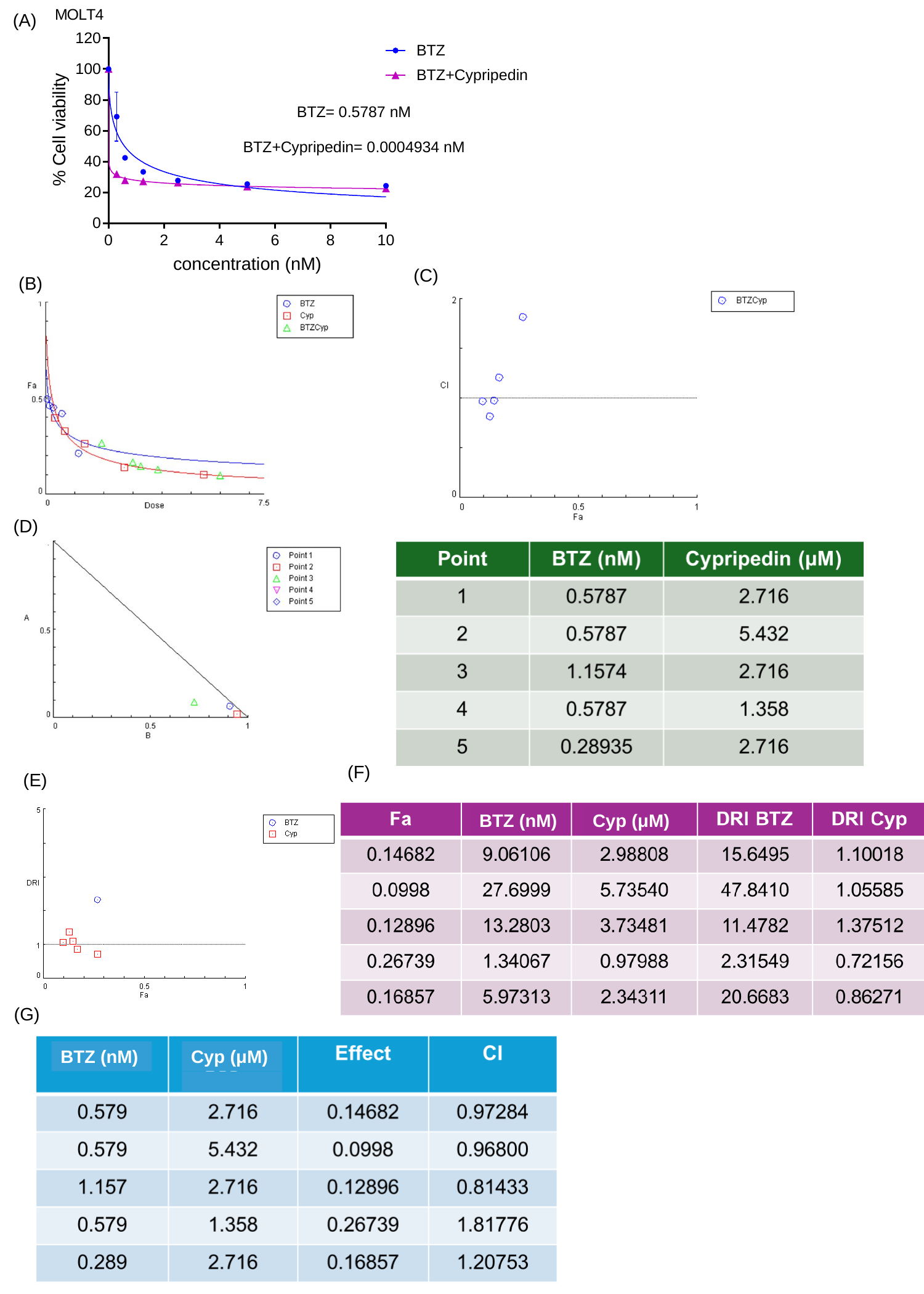

(A)
(C)
(B)
(D)
(F)
(E)
BTZ (nM)
Cyp (μM)
(G)
BTZ (nM)
Cyp (μM)

## Slide 6
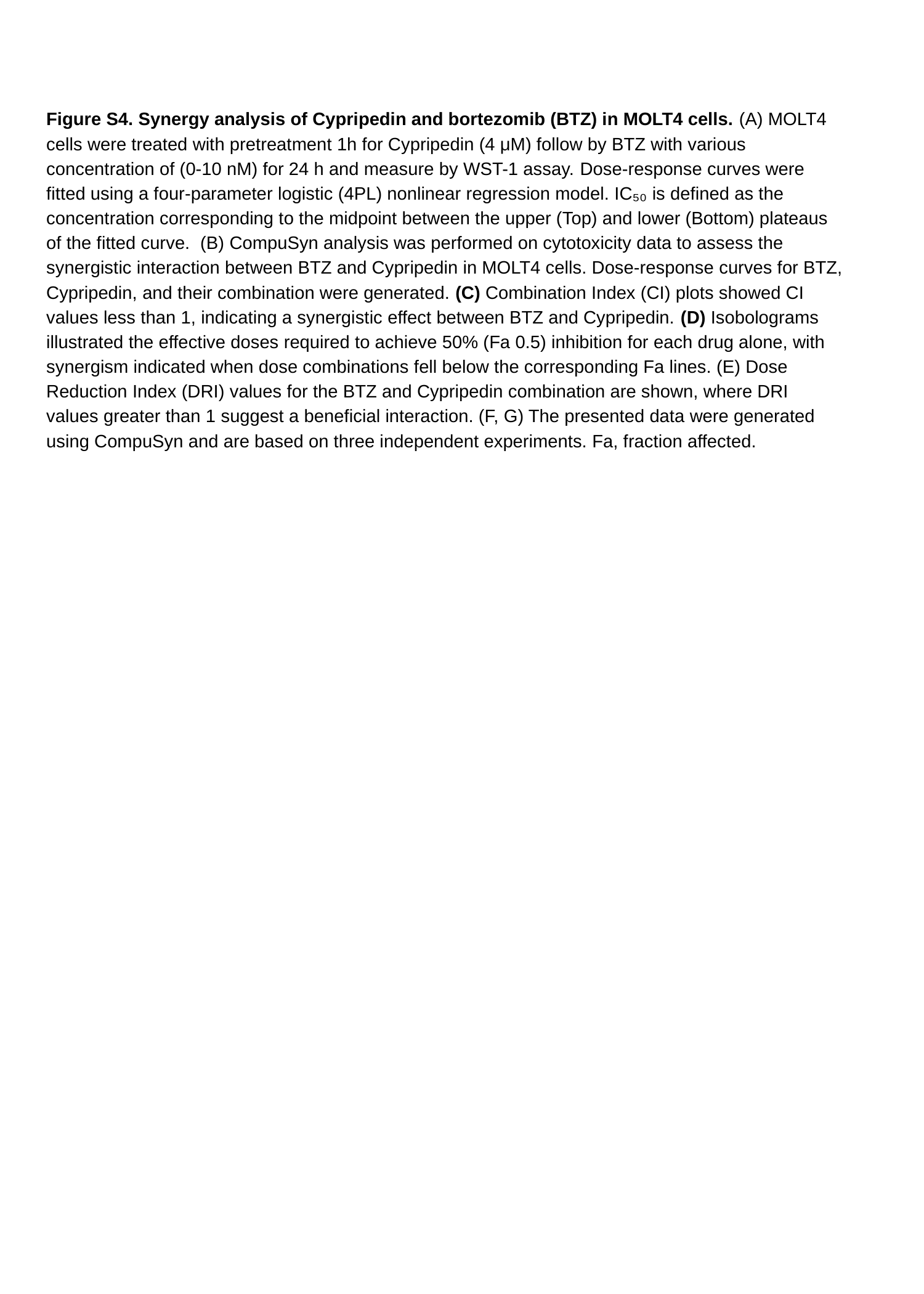

Figure S4. Synergy analysis of Cypripedin and bortezomib (BTZ) in MOLT4 cells. (A) MOLT4 cells were treated with pretreatment 1h for Cypripedin (4 μM) follow by BTZ with various concentration of (0-10 nM) for 24 h and measure by WST-1 assay. Dose-response curves were fitted using a four-parameter logistic (4PL) nonlinear regression model. IC₅₀ is defined as the concentration corresponding to the midpoint between the upper (Top) and lower (Bottom) plateaus of the fitted curve. (B) CompuSyn analysis was performed on cytotoxicity data to assess the synergistic interaction between BTZ and Cypripedin in MOLT4 cells. Dose-response curves for BTZ, Cypripedin, and their combination were generated. (C) Combination Index (CI) plots showed CI values less than 1, indicating a synergistic effect between BTZ and Cypripedin. (D) Isobolograms illustrated the effective doses required to achieve 50% (Fa 0.5) inhibition for each drug alone, with synergism indicated when dose combinations fell below the corresponding Fa lines. (E) Dose Reduction Index (DRI) values for the BTZ and Cypripedin combination are shown, where DRI values greater than 1 suggest a beneficial interaction. (F, G) The presented data were generated using CompuSyn and are based on three independent experiments. Fa, fraction affected.

## Slide 7
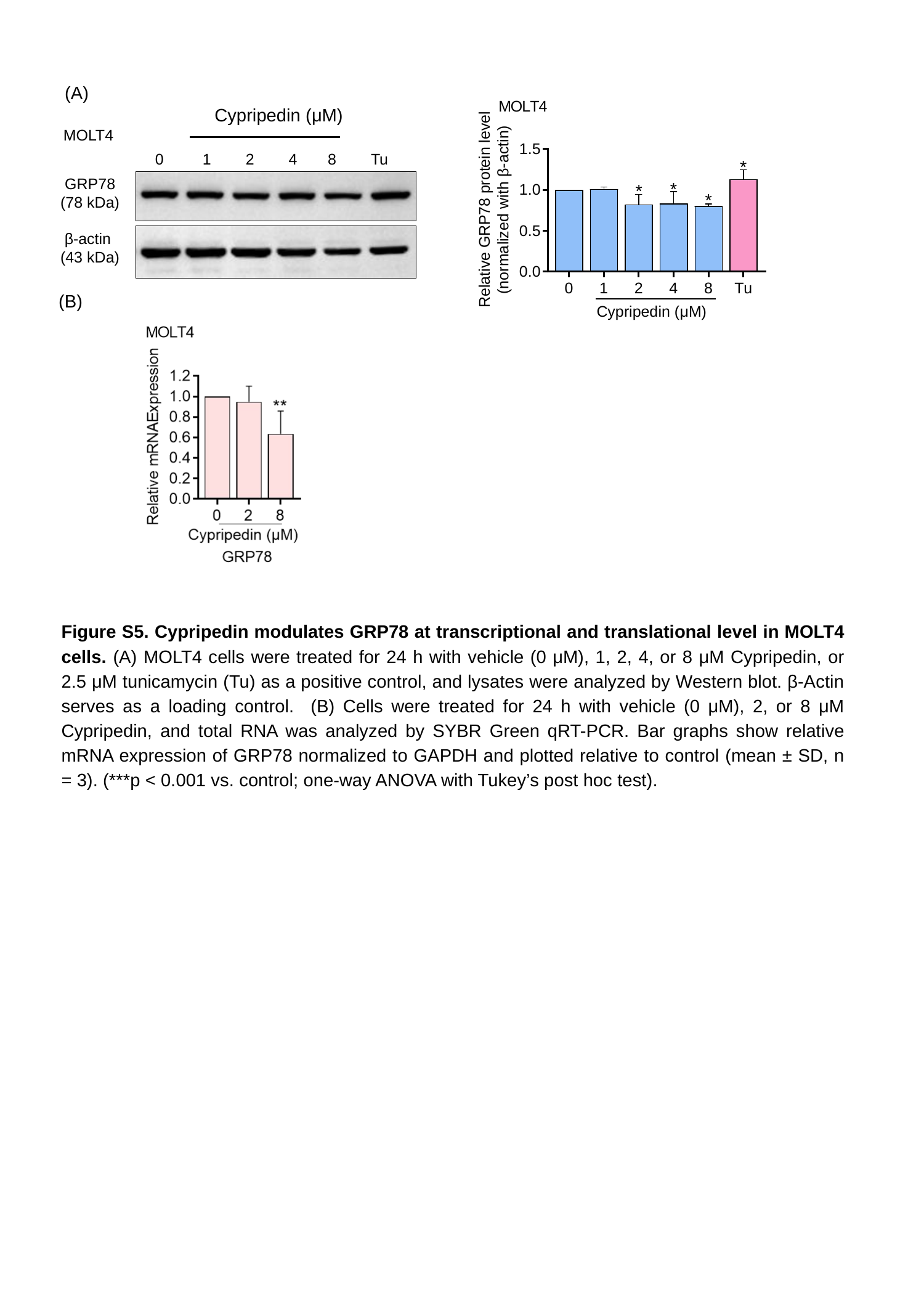

(A)
Cypripedin (μM)
MOLT4
     0      1       2        4       8     Tu
GRP78 (78 kDa)
β-actin
(43 kDa)
(B)
Figure S5. Cypripedin modulates GRP78 at transcriptional and translational level in MOLT4 cells. (A) MOLT4 cells were treated for 24 h with vehicle (0 μM), 1, 2, 4, or 8 μM Cypripedin, or 2.5 μM tunicamycin (Tu) as a positive control, and lysates were analyzed by Western blot. β-Actin serves as a loading control. (B) Cells were treated for 24 h with vehicle (0 μM), 2, or 8 μM Cypripedin, and total RNA was analyzed by SYBR Green qRT-PCR. Bar graphs show relative mRNA expression of GRP78 normalized to GAPDH and plotted relative to control (mean ± SD, n = 3). (***p < 0.001 vs. control; one-way ANOVA with Tukey’s post hoc test).

## Slide 8
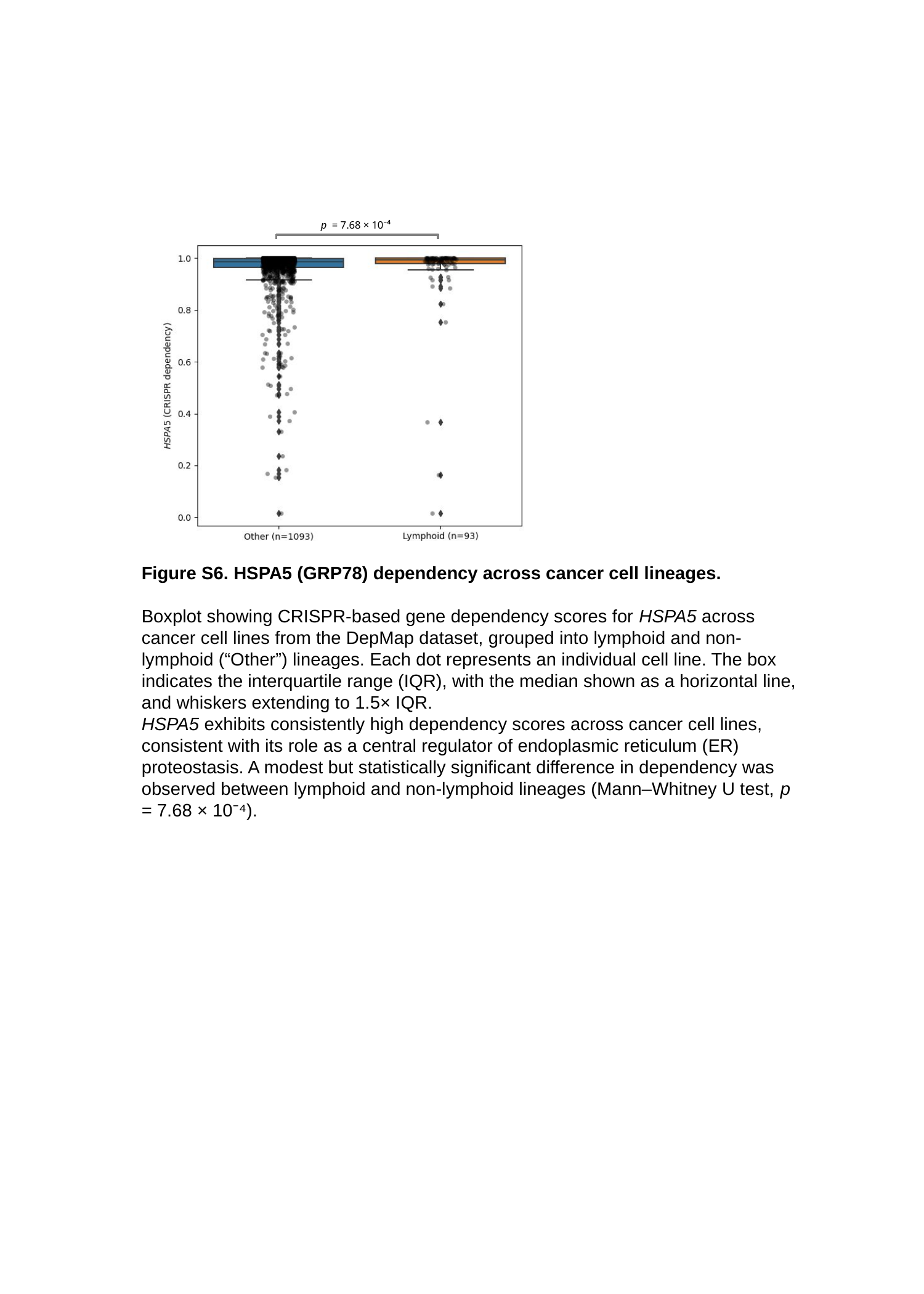

p = 7.68 × 10⁻⁴
Figure S6. HSPA5 (GRP78) dependency across cancer cell lineages.
Boxplot showing CRISPR-based gene dependency scores for HSPA5 across cancer cell lines from the DepMap dataset, grouped into lymphoid and non-lymphoid (“Other”) lineages. Each dot represents an individual cell line. The box indicates the interquartile range (IQR), with the median shown as a horizontal line, and whiskers extending to 1.5× IQR.
HSPA5 exhibits consistently high dependency scores across cancer cell lines, consistent with its role as a central regulator of endoplasmic reticulum (ER) proteostasis. A modest but statistically significant difference in dependency was observed between lymphoid and non-lymphoid lineages (Mann–Whitney U test, p = 7.68 × 10⁻⁴).
