# Supplementary material for: Cypripedin Induces Apoptosis and Synergizes with Bortezomib via ER Stress Mediated Ubiquitination of GRP78 in T-Cell Acute Lymphoblastic Leukemia
Source: Molecules. 2026 May 25;31(11):1823. doi: 10.3390/molecules31111823 (PMC13257771; doi:10.3390/molecules31111823)
Supplement: Supplementary file 1 [file molecules-31-01823-s001.zip › Supplementary Figures S7 and S8.pdf]

Supplementary Figure S7—Raw data for Figure 3 (A–G) and CompuSyn Report.

Figure 3 (A)

nM	BTZ			BTZ+Cypripedin (4 μM)		
X	A:Y1	A:Y2	A:Y3	B:Y1	B:Y2	B:Y3
0.000	100.0000	100.0000	100.0000	100.0000	100.0000	100.0000
3.250	82.47803	78.94701	79.08799	60.79509	56.51016	54.92782
6.510	60.08900	65.71134	57.20690	42.22340	47.48436	51.73221
13.010	28.42778	33.63626	34.67826	22.88972	30.91421	26.99602
26.030	18.95561	23.91668	23.13544	12.80052	19.24350	20.07466
52.050	13.91393	19.17592	16.69837	7.962668	13.46243	11.78447
	N1	N2	N3	N1	N2	N3

% Cell Viability

Figure 3(B-G)

BTZ(nM)	N1	N2	N3	Ave
0	0.8645	0.873025	0.929388	0.888971
1.12	0.699275	0.726175	0.764	0.729817
2.23	0.6837	0.6994	0.768263	0.717121
4.46	0.62795	0.6034	0.775813	0.669054
8.93	0.35845	0.385188	0.42385	0.389163
17.85	0.17785	0.235925	0.258	0.223925
**Cypripedin(uM)**				
0	0.83825	0.845263	0.910038	0.864517
0.816	0.7093	0.706575	0.76285	0.726242
1.631	0.7049	0.67435	0.710571	0.696607
3.262	0.60565	0.578688	0.61355	0.599296
6.524	0.4756	0.470288	0.509163	0.485017
13.048	0.428325	0.403063	0.363938	0.398442
**Combination**				
0	0.8447	0.841963	0.981175	0.889279
1	0.246975	0.229113	0.227625	0.234571
2	0.174225	0.224013	0.222013	0.20675
3	0.166525	0.16705	0.140338	0.157971
4	0.2166	0.226163	0.295571	0.246111
5	0.344883	0.387629	0.385304	0.372606

	BTZ(nM)	Cypripedine (uM)
1	8.93	6.52
2	8.93	13.04
3	17.85	6.52
4	8.93	3.26
5	4.46	6.52

	%Cell Viability		
BTZ(nM)	N1	N2	N3
0	100	100	100
1.12	80.8878	83.17918	82.20468
2.23	79.08618	80.11225	82.66331
4.46	72.63736	69.116	83.47568
8.93	41.46327	44.12102	45.6053
17.85	20.57259	27.02385	27.76022
Cypripedin(uM)			
0	100	100	100
0.816	84.61676	83.59238	83.82622
1.631	84.09186	79.77995	78.08149
3.262	72.25171	68.46246	67.4203
6.524	56.73725	55.63804	55.94962
13.048	51.09752	47.68489	39.99148
Combination			
0	100	100	100
1	29.23819	27.21172	23.19923
2	20.62567	26.60599	22.62721
3	19.7141	19.84055	14.303
4	25.64224	26.86135	30.12417
5	40.82909	46.03877	39.26967

CompuSyn Report

Experiment Name: Jurkat
Date:
File Name: C:\Users\Admin\Desktop\May 2025_MS Cypripedin_Zin\Compusym results\1.cse
Description

Drug: Bortezomib (BTZ) [nM]
Drug: Cypripedin (Cyp) [uM]
Drug Combo: Bortezomib Cypripedin (BTZCyp) (BTZ+Cyp)

Data for Drug: BTZ [nM]

Dose	Effect
1.12	0.72982
2.23	0.71712
4.46	0.66905
8.93	0.38916
17.85	0.22393

5 data points entered.
X-int: 0.74579
Y-int: 0.63079 +/- 0.14355
m: -0.8458 +/- 0.18483
Dm: 5.56921
r: -0.9352

Data for Drug: Cyp [uM]

Dose	Effect
0.816	0.72624
1.631	0.69661
3.262	0.5993
6.524	0.48502
13.048	0.39844

5 data points entered.
X-int: 0.79880
Y-int: 0.42260 +/- 0.03047
m: -0.5290 +/- 0.04569
Dm: 6.29210
r: -0.9890

Data for Non-Constant Combo: BTZCyp (BTZ+Cyp)

Dose BTZ	Dose Cyp	Effect
8.93	6.52	0.23457
8.93	13.04	0.20675
17.85	6.52	0.15797
8.93	3.26	0.24611

Dose BTZ	Dose Cyp	Effect
4.46	6.52	0.37261

5 data points entered.

Dose-Effect Curve

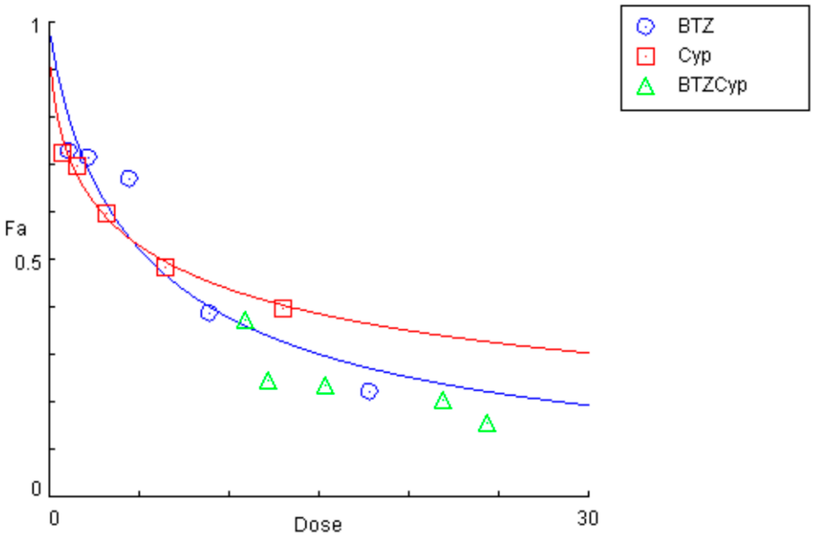

Median-Effect Plot

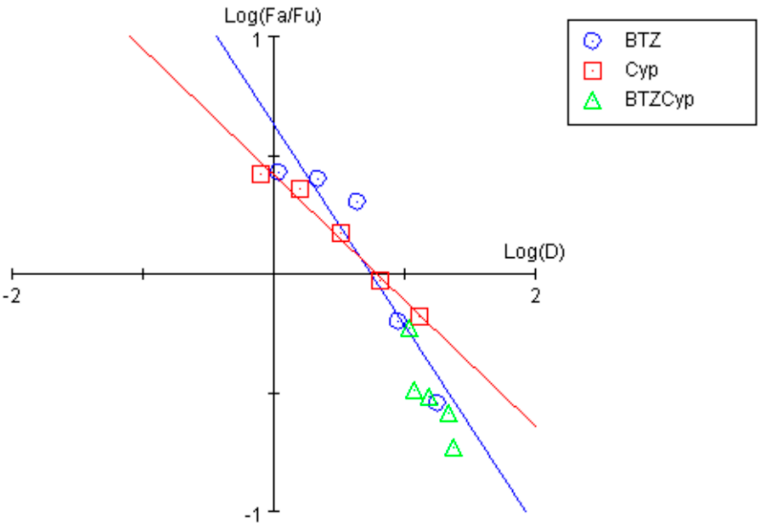

CI Data for Non-Constant Combo: BTZCyp (BTZ+Cyp)

Dose BTZ	Dose Cyp	Effect	CI
8.93	6.52	0.23457	0.50689
8.93	13.04	0.20675	0.49024
17.85	6.52	0.15797	0.48702
8.93	3.26	0.24611	0.48925

Dose BTZ	Dose Cyp	Effect	CI
4.46	6.52	0.37261	0.81954

Combination Index Plot

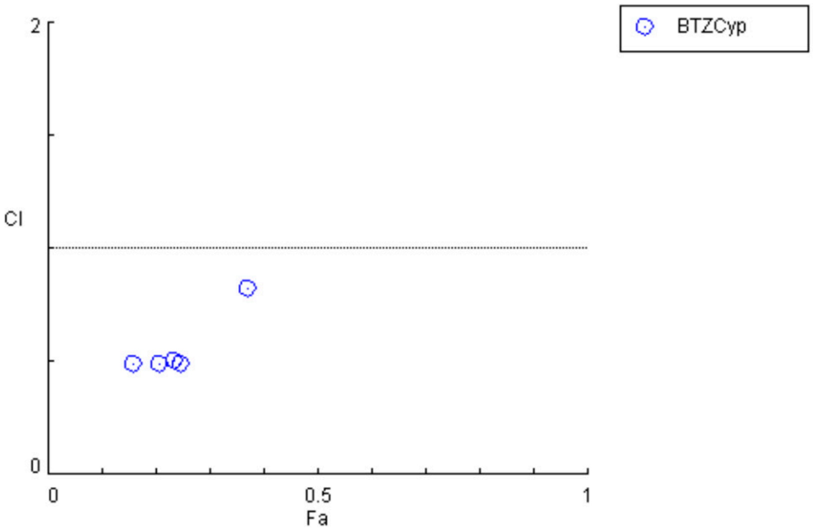

Logarithmic Combination Index Plot

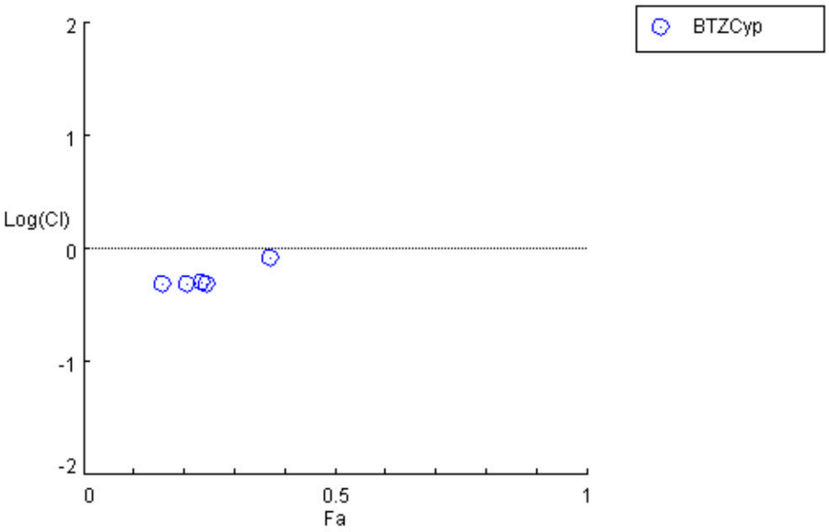

DRI Data for Non-Constant Combo: BTZCyp (BTZ+Cyp)

Fa	Dose BTZ	Dose Cyp	DRI BTZ	DRI Cyp
0.23457	22.5461	58.8380	2.52476	9.02424
0.20675	27.3040	79.9094	3.05755	6.12802
0.15797	40.2761	148.763	2.25636	22.8164
0.24611	20.9224	52.2113	2.34293	16.0157
0.37261	10.3117	16.8466	2.31205	2.58384

DRI Plot for Non-Constant Combo: BTZCyp (BTZ+Cyp)

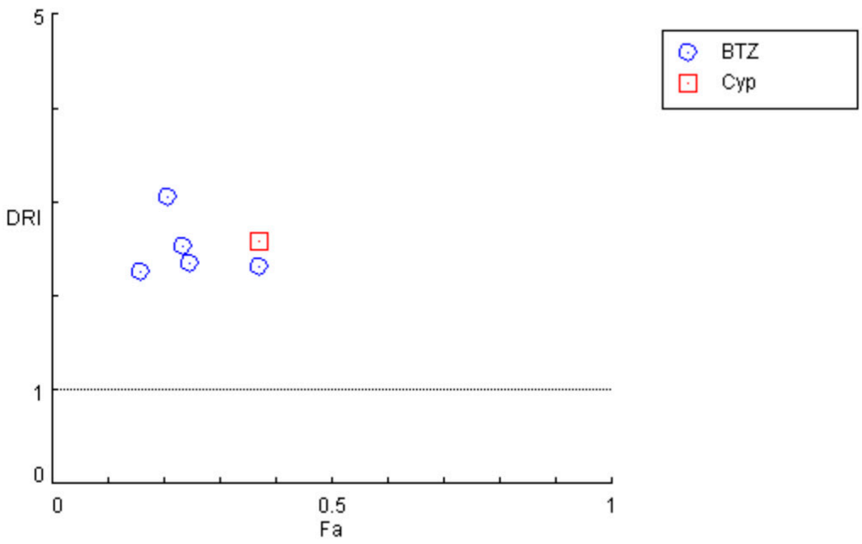

Log(DRI) Plot for Non-Constant Combo: BTZCyp (BTZ+Cyp)

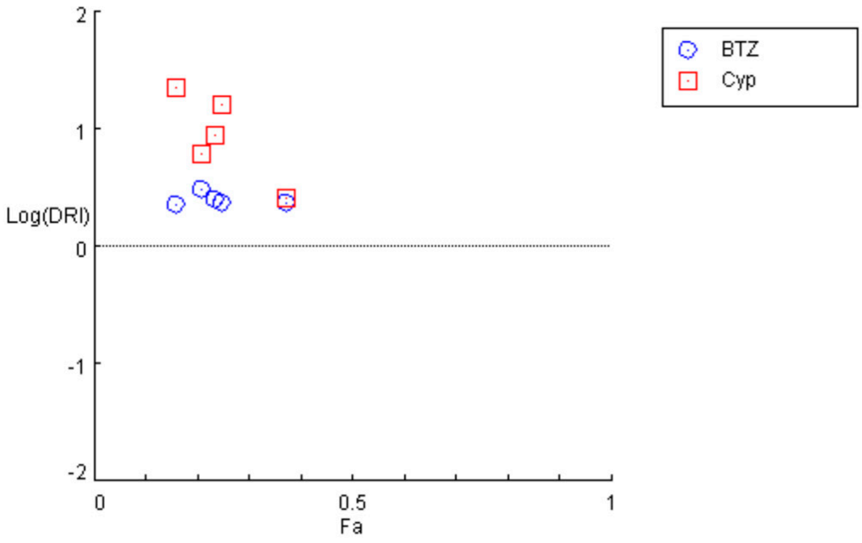

Normalized Isobologram for Combo: BTZCyp (BTZ+Cyp)

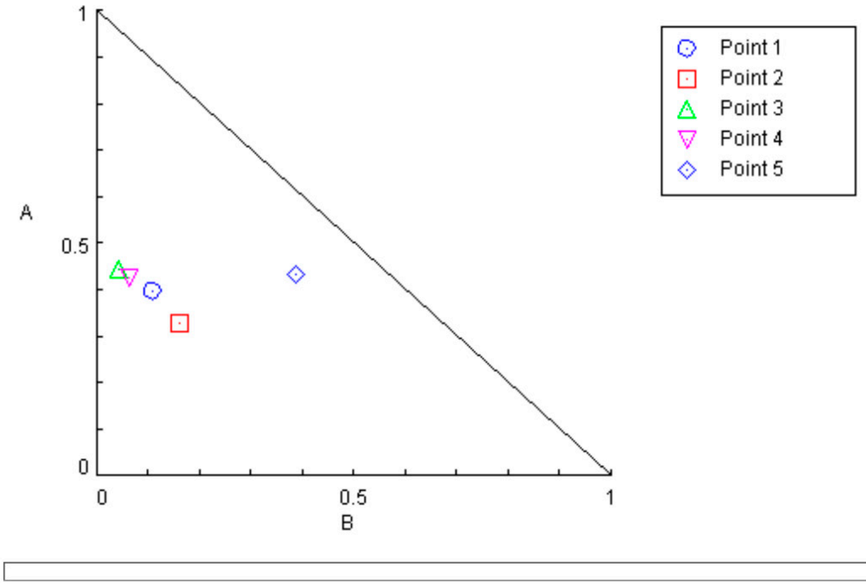

Supplementary Figure S8 – Overlayed Blots, whole membrane.

Cypripedin Induces Apoptosis and Synergizes with Bortezomib via ER Stress Mediated Ubiquitination of GRP78 in T-cell acute lymphoblastic leukemia

Overlay raw data for Western Blot Analysis

Figure 4

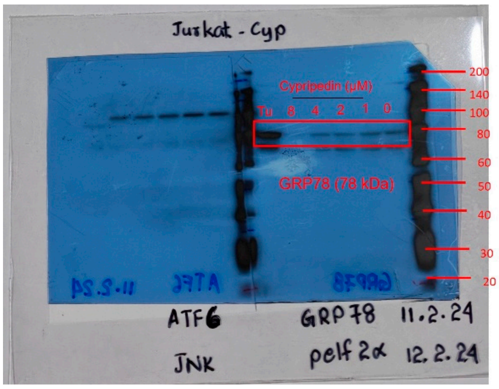

Exposure time for GRP78 - 3 min

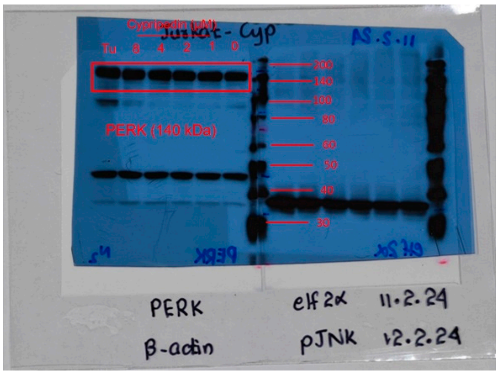

Exposure time for PERK - 1 min

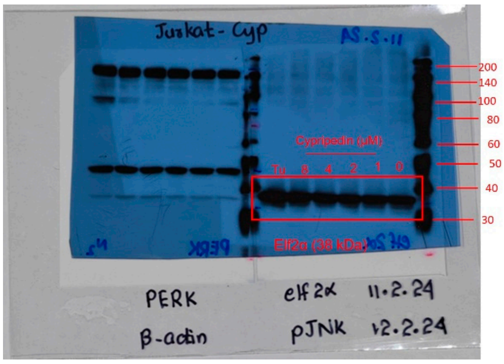

Exposure time for elf2α - 1 min

Supplementary Figure S8 – Overlayed Blots, whole membrane (Cont.)

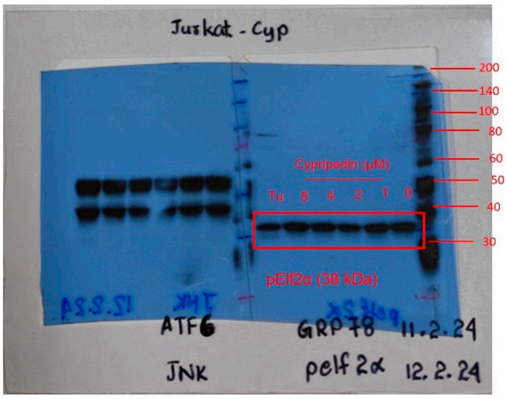

Exposure time for pelf2α - 2 min

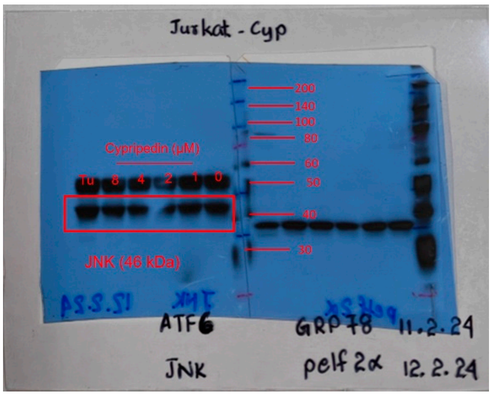

Exposure time for JNK- 2 min

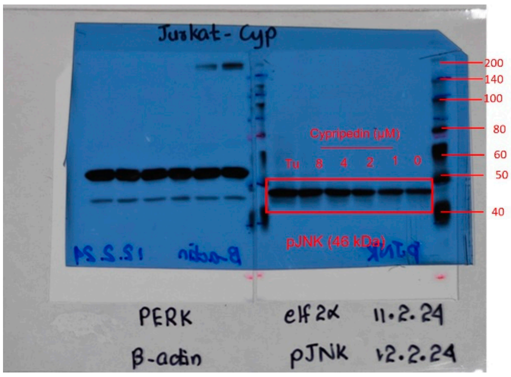

Exposure time for pJNK- 2 min

Supplementary Figure S8 – Overlayed Blots, whole membrane (Cont.)

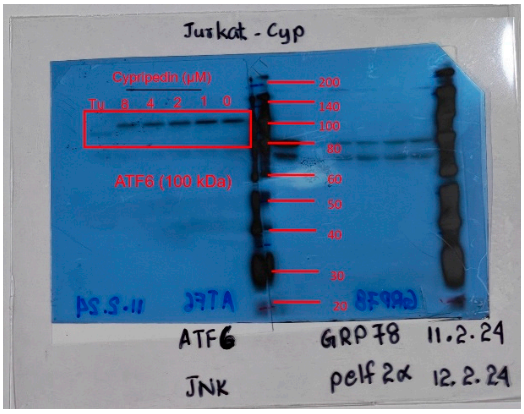

Exposure time for ATF6 - 3 min

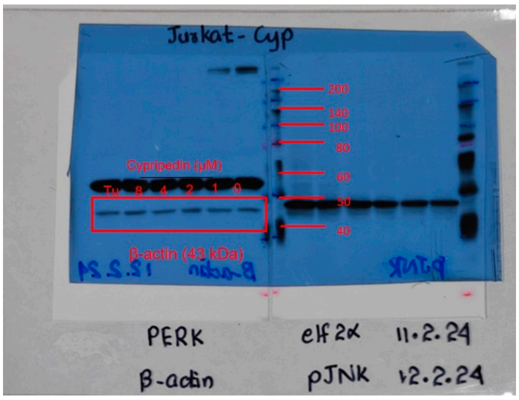

Exposure time for β-actin- 2 min

For Figure 4, protein detection was performed using a traditional chemiluminescence method in a dark room, followed by exposure to X-ray film. Two types of protein markers are a dual-color ladder and a biotinylated ladder that were loaded in the same lane during SDS-PAGE. Because the dual-color ladder is not visible on X-ray film, the biotinylated ladder was included to allow molecular weight visualization on the developed film. The dual-color ladder was visible on both the gel and membrane, whereas the biotinylated ladder was detectable only on the film. The protein markers used were Precision Plus Protein Dual Color Standards (#1610374) and the Biotinylated Protein Ladder Detection Pack (#7727). After film development, images were digitized using a scanner.

Supplementary Figure S8 – Overlayed Blots, whole membrane (Cont.)

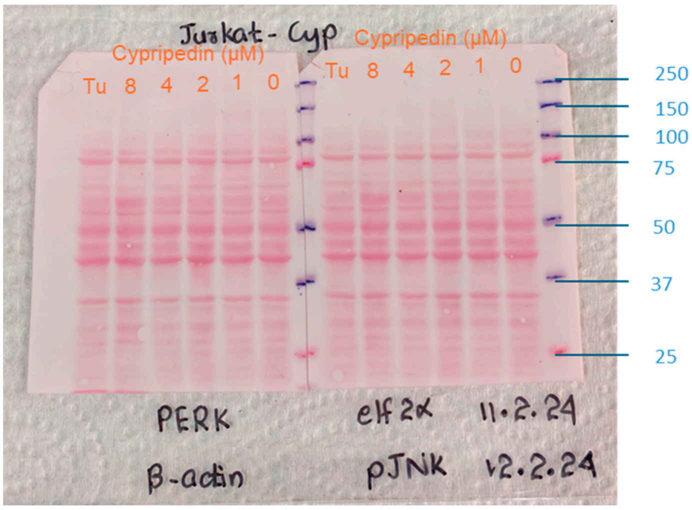

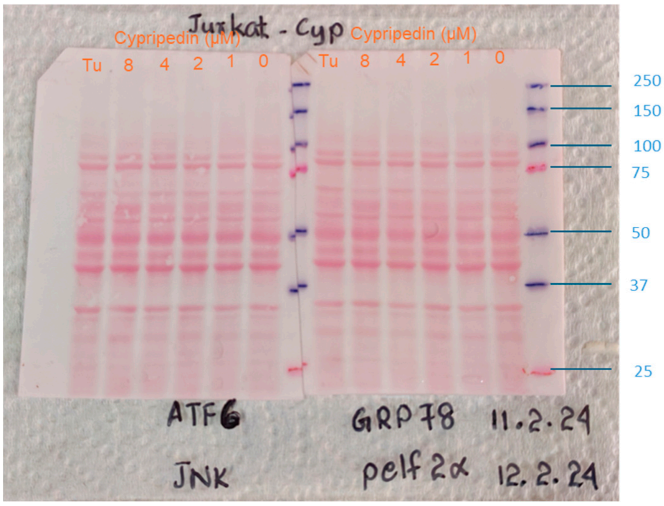

The Western blot membrane shown in Figure 4 was stained with Ponceau S to visualize and confirm the uniform transfer and localization of total proteins on the membrane prior to immunodetection.

Figure 5

File name for Ub-GRP78 membrane

Supplementary Figure S8 – Overlayed Blots, whole membrane (Cont.)

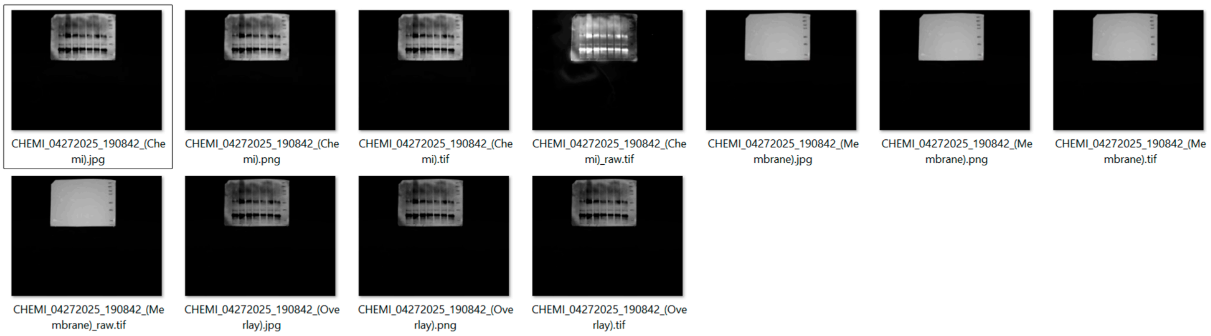

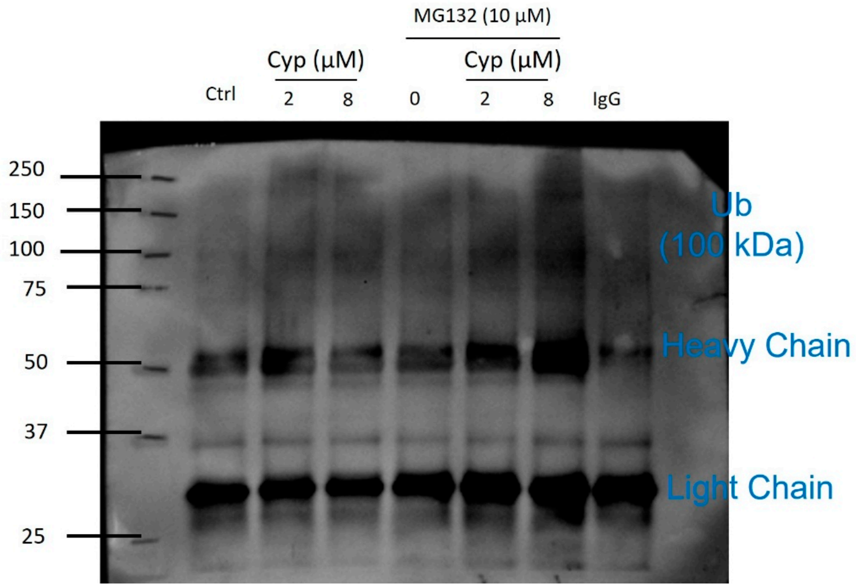

Exposure time- 3 min

File name for β-actin membrane

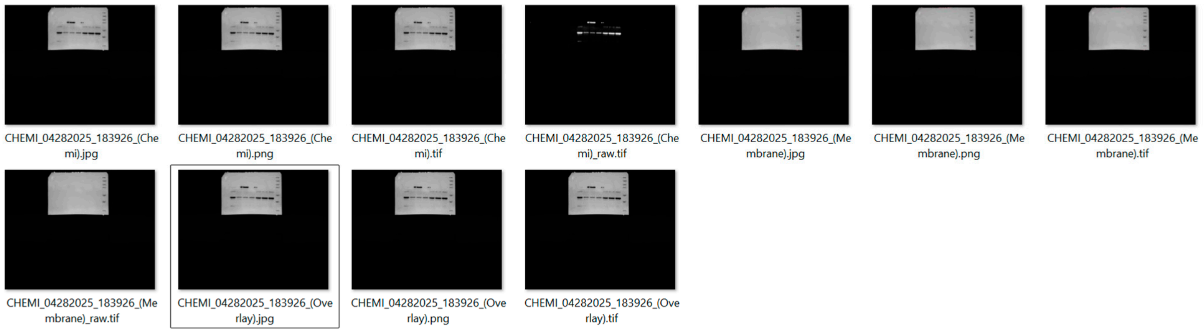

Supplementary Figure S8 – Overlayed Blots, whole membrane (Cont.)

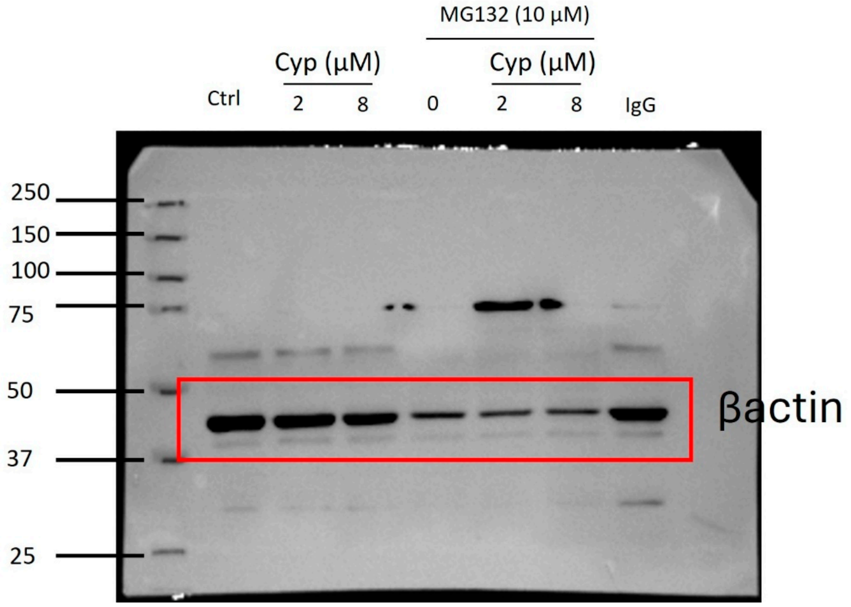

Exposure time- 1 min

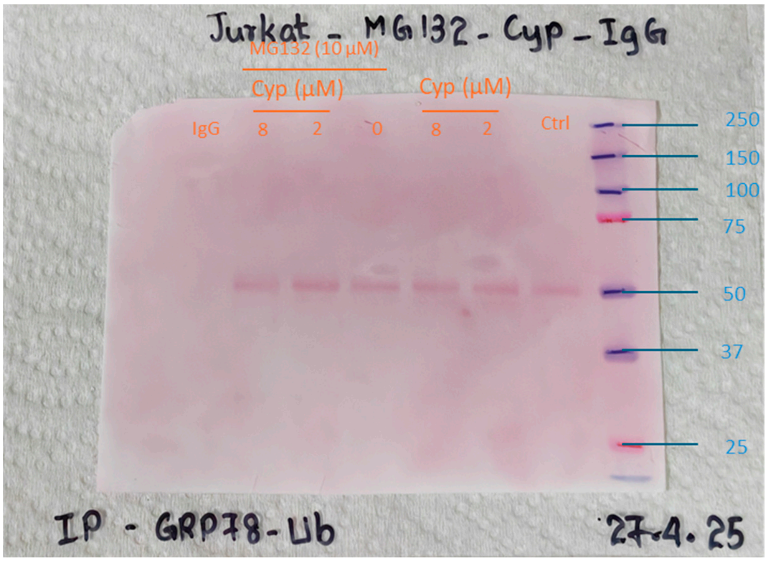

The Immunoprecipitation for Ub-GRP78 shown in Figure 5 was stained with Ponceau S to visualize and confirm the uniform transfer and localization of total proteins on the membrane prior to immunodetection.

Supplementary Figure S8 – Overlayed Blots, whole membrane (Cont.)

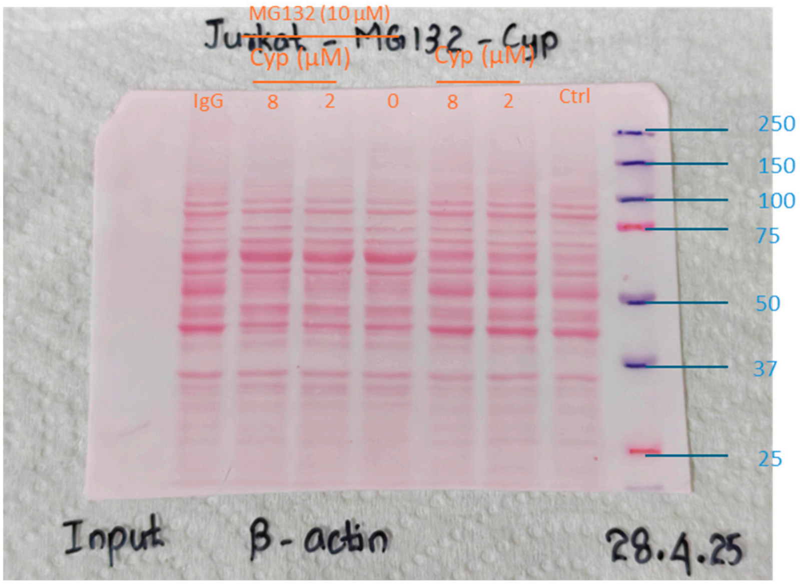

The Immunoprecipitation for Input (β-actin) shown in Figure 5 was stained with Ponceau S to visualize and confirm the uniform transfer and localization of total proteins on the membrane prior to immunodetection.

For Figure 5, protein detection was performed using a iBright CL1500 Imaging Systems chemiluminescence method. The protein markers used were Precision Plus Protein Dual Color Standards (#1610374).
